# The *Globodera pallida* SPRYSEC Effector *Gp*SPRY-414-2 That Suppresses Plant Defenses Targets a Regulatory Component of the Dynamic Microtubule Network

**DOI:** 10.3389/fpls.2018.01019

**Published:** 2018-07-12

**Authors:** Yuanyuan Mei, Kathryn M. Wright, Annelies Haegeman, Lander Bauters, Amalia Diaz-Granados, Aska Goverse, Godelieve Gheysen, John T. Jones, Sophie Mantelin

**Affiliations:** ^1^Dundee Effector Consortium, Cell and Molecular Sciences Group, The James Hutton Institute, Dundee, United Kingdom; ^2^Faculty of Bioscience Engineering, Department of Biotechnology, Ghent University, Ghent, Belgium; ^3^Laboratory of Nematology, Department of Plant Sciences, Wageningen University, Wageningen, Netherlands; ^4^School of Biology, University of St Andrews, St Andrews, United Kingdom

**Keywords:** CLASP, defense suppression, effector, microtubules, nematode, potato, SPRYSEC

## Abstract

The white potato cyst nematode, *Globodera pallida*, is an obligate biotrophic pathogen of a limited number of Solanaceous plants. Like other plant pathogens, *G. pallida* deploys effectors into its host that manipulate the plant to the benefit of the nematode. Genome analysis has led to the identification of large numbers of candidate effectors from this nematode, including the cyst nematode-specific SPRYSEC proteins. These are a secreted subset of a hugely expanded gene family encoding SPRY domain-containing proteins, many of which remain to be characterized. We investigated the function of one of these SPRYSEC effector candidates, *Gp*SPRY-414-2. Expression of the gene encoding *Gp*SPRY-414-2 is restricted to the dorsal pharyngeal gland cell and reducing its expression in *G. pallida* infective second stage juveniles using RNA interference causes a reduction in parasitic success on potato. Transient expression assays in *Nicotiana benthamiana* indicated that *Gp*SPRY-414-2 disrupts plant defenses. It specifically suppresses effector-triggered immunity (ETI) induced by co-expression of the *Gpa2* resistance gene and its cognate avirulence factor *RBP-1*. It also causes a reduction in the production of reactive oxygen species triggered by exposure of plants to the bacterial flagellin epitope flg22. Yeast two-hybrid screening identified a potato cytoplasmic linker protein (CLIP)-associated protein (*St*CLASP) as a host target of *Gp*SPRY-414-2. The two proteins co-localize *in planta* at the microtubules. CLASPs are members of a conserved class of microtubule-associated proteins that contribute to microtubule stability and growth. However, disruption of the microtubule network does not prevent suppression of ETI by *Gp*SPRY-414-2 nor the interaction of the effector with its host target. Besides, *Gp*SPRY-414-2 stabilizes its target while effector dimerization and the formation of high molecular weight protein complexes including *Gp*SPRY-414-2 are prompted in the presence of the *St*CLASP. These data indicate that the nematode effector *Gp*SPRY-414-2 targets the microtubules to facilitate infection.

## Introduction

The white potato cyst nematode, *Globodera pallida*, is a sedentary root endoparasite that causes yield losses on Solanaceous crops worldwide (Jones et al., 2013, CABI Invasive Species Compendium http://www.cabi.org/isc/datasheet/27033). It invades host plants in the elongation zone behind the root tip and then migrates through the inner cortex layers to initiate a feeding site near the vascular tissues (Lilley et al., 2005). The specialized feeding site, or syncytium, is a large multinucleate cell formed by the breakdown of plant cell walls and subsequent fusion of adjacent protoplasts (Kyndt et al., 2013). *G. pallida is* an obligate biotroph and relies on the syncytium for all nutrients required for its growth and reproduction.

The success of colonization by biotrophic pathogens is dependent upon their modulation of plant defenses. These can be classified into two different branches, pathogen-associated molecular patterns (PAMP)-triggered immunity (PTI). and effector-triggered immunity (ETI) (Dodds and Rathjen, 2010). Perception of PAMPs, such as the bacterial flagellin derivative peptide flg22, provokes a range of downstream responses that include production of reactive oxygen species (ROS) to ward off pathogen attack. Recently, an ascaroside (ascr#18) has been identified as a PAMP from nematodes which can induce PTI in plants (Manosalva et al., 2015). Adapted biotrophic pathogens release effectors that suppress PTI. In the second layer of plant defenses, the products of resistance (*R*) genes recognize the presence of some of these effectors to trigger ETI. Effectors that are recognized are the products of avirulence (*Avr*) genes. Detection of these avirulence factors frequently results in a hypersensitive reaction (HR) involving a local and controlled plant programmed cell death that contains the pathogen. Like other biotrophic pathogens, *G. pallida* has to suppress plant defenses in order to survive. It is widely accepted that both suppression of plant defenses and successful establishment, as well as maintenance of feeding sites, are mediated by effector proteins, many of which are produced in the nematode pharyngeal gland cells and secreted into the plant through the stylet (Gheysen and Jones, 2006; Haegeman et al., 2012).

A large number of effector proteins have been identified from the genome sequence of *G. pallida* (Cotton et al., 2014; Thorpe et al., 2014). Of particular note is a large family of SP1a and Ryanodine receptor (SPRY) domain-containing proteins. Some of these are predicted to be secreted proteins (SPRYSECs) that are produced within the dorsal gland cell of stage 2 infective juveniles (J2s) and are thought to function as effectors (Mei et al., 2015). One member of this gene family, *RAN-binding protein-1* (*RBP-1*), induces an HR in *Nicotiana benthamiana* leaves when co-expressed with its cognate *R*-gene, the potato coiled-coil nucleotide-binding leucine-rich repeat (CC-NB-LRR) resistance protein-encoding gene *Gpa2* (Sacco et al., 2009). A similar gene family of SPRYSEC effectors has been described from *Globodera rostochiensis* (Eves-van den Akker et al., 2016). One *G. rostochiensis* SPRYSEC (SPRYSEC-19) physically interacts with SW5F, a CC-NB-LRR type disease resistance protein (Rehman et al., 2009), and was subsequently shown to suppress the ETI-dependent programmed cell death mediated by several CC-NB-LRR immune receptors (Postma et al., 2012). The size of this predicted *SPRY* gene family from *G. pallida* together with the diverse subcellular localization patterns of the secreted members suggest that the proteins encoded by *SPRYSECs* may play various roles in the interaction between nematode and host plant (Jones et al., 2009; Diaz-Granados et al., 2016). Characterizing the functions of other members of the *SPRYSEC* gene family will be of great help in unraveling their roles in plant parasitism.

Here we describe the functional characterization of the SPRYSEC protein *Gp*SPRY-414-2 from *G. pallida*. We investigated the role of *Gp*SPRY-414-2 in suppressing plant defenses. Through yeast two-hybrid (Y2H) screening and *in planta* assays this effector protein was shown to interact with a potato cytoplasmic linker protein (CLIP)-associated protein (CLASP). CLASPs are members of a conserved class of microtubule-associated proteins (MAPs; Gardiner, 2013) that localize on the plus-end of microtubules and that have various functions in cell motility, mitosis and defense (e.g., Quentin et al., 2016). CLASPs are needed for microtubule cytoskeleton polarization and contribute to microtubule stability and growth (Al-Bassam et al., 2010). Studies also indicate that CLASP1 may control some aspects of auxin transport by interacting with retromer component sorting nexin 1 (SNX1; Ambrose et al., 2013). A drastic distortion of microtubule organization (De Almeida Engler et al., 2004) and manipulation of the host's auxin response and transport (Grunewald et al., 2009) have been previously shown to be required for nematode infection. Our data indicate that the nematode effector *Gp*SPRY-414-2 targets the microtubules to facilitate infection.

## Materials and methods

### *GpSPRY-414-2, GpSPRYSEC* control genes and *StCLASP* cloning

Previous work has identified a number of *SPRYSEC* effector genes within the family encoding SPRY domain proteins in *G. pallida* (Mei et al., 2015). One of these effectors, *GpSPRY-414-2* (GenBank reference MH003298; related to GPLIN_000195600), was selected for further study due to its abundance in the J2 transcriptome. As in previous work, the *Globodera pallida* nematodes used in the present study are from the standard Pa2/3 population “Lindley” (Phillips and Trudgill, 1998). Nematodes were maintained on susceptible potato (*Solanum tuberosum*) cv. Désirée in a greenhouse. Cysts were extracted using standard protocols (Caswell et al., 1985) and stored at 4°C for at least 6 months before use. Second-stage juveniles (J2) were hatched in tomato root diffusate prepared as previously described (Jones et al., 1996).

The full-length coding sequence of *GpSPRY-414-2* was PCR amplified without the predicted signal peptide from cDNA of J2s (prepared as in Mei et al., 2015), using gene specific primers including a leader sequence (ACCATG) for translation (Supplementary Table 1). PCR was performed using the proof reading KOD DNA polymerase (Novagen) according to the manufacturer's instructions and amplification products of the expected size (639 bp) were purified from 1.5% (w/v) agarose gels using the QIAquick Gel Extraction Kit (Qiagen) and inserted into the pCR8/GW/TOPO Gateway ENTRY vector by TA cloning following the manufacturer's instructions (Life Technologies). The effector clone was subsequently recombined into the Gateway-compatible binary expression vectors pK7WGF2/pH7WGR2 and pK7FWG2/pH7RWG2 for eGFP/mRFP N-terminal and C-terminal fusions respectively (Karimi et al., 2002), the pCL113 vector (YFPc domain; Bos et al., 2010) to create a N-terminal split-YFP fusion for bimolecular fluorescence complementation (BiFC) assays or the pDEST32 vector to generate a N-terminal fusion with the GAL4 DNA-binding domain for Y2H screening (Life Technologies ProQuest™ Two-Hybrid System). LR clonase (Life Technologies) was used for all recombination reactions following the manufacturer's instructions.

Similarly, three other *SPRYSEC* candidate effector genes, previously cloned in pCR8/GW/TOPO without their endogenous signal peptides (Mei et al., 2015), were recombined into the yeast bait vector pDEST32: *GpSPRY-24D4* (GenBank reference MH003299; related to GPLIN_001465500), *GpSPRY-17I9-1* (GenBank reference MH003301), and *GpSPRY-12N3* (GenBank reference MH003300). In addition, *GpSPRY-24D4* was also cloned into the eGFP/mRFP localization vectors as well as in the split-YFP vector pCL113.

A Y2H screen (below) identified a potato *St*CLASP as putative target of *Gp*SPRY-414-2. The full-length coding sequence of the *StCLASP* gene was cloned from potato cultivar Désirée cDNA using gene-specific primers (Supplementary Table 1) that were designed based on the available genomic gene sequence (potato Phureja DM1-3 sequence located on superscaffold PGSC0003DMB000000115; later annotated as gene LOC102584952, coding sequence GenBank accession XM_006350231.2). The coding sequence of the gene was originally identified based on AUGUSTUS gene finding tool prediction (Stanke et al., 2008) and on similarity to the orthologous sequence from tomato which was annotated (Solyc09g063030). PCR was performed using the proof-reading KOD DNA polymerase (Novagen) and products were resolved on 1% (w/v) agarose gels before purification with the QIAquick Gel Extraction Kit (Qiagen) following the manufacturer's instructions. The 4,290 bp coding sequence was subcloned into pCR8/GW/TOPO and then recombined into expression vectors as described above except that *StCLASP* (full length construct later called *StCLASP-FL*) was cloned into the split-YFP vector pCL112 (YFPn domain; Bos et al., 2010) and in the prey vector pDEST22 (Life Technologies) to generate a fusion with the GAL4 activation domain for the Y2H assay. Finally, the truncated version of *StCLASP*, as found in Y2H prey clone G1-5 (GenBank reference MH003297) and later called *StCLASP-Y2H*, was subcloned into the Gateway ENTRY vector pDONR221 by BP reaction from the pDEST22-G1-5 clone following the manufacturer's instructions (Life Technology) and recombined into N-terminal fusion vectors pK7WGF2, pH7WGR2, and pCL112.

The domain architecture of the SPRYSEC effectors and CLASP proteins was analyzed using the Simple Modular Architecture Research Tool (SMART; Schultz et al., 1998). The integrity of the gene sequence for each construct as well as the reading frame for the fusions were confirmed by Sanger sequencing (Primers in Supplementary Table 1). The resulting binary and yeast vectors were transformed into *Agrobacterium tumefaciens* strain GV3101 carrying the helper vector pBBR1MCS5-VIRG-N54D encoding the virG^N54D^ virulence factor (van der Fits et al., 2000) and yeast Mav203 cells, respectively.

### Silencing of *GpSPRY-414-2* in *G. pallida* by RNA interference

A 263 bp fragment of the *GpSPRY-414-2* clone targeting nucleotides 157 to 419 was selected for silencing. A fragment of GFP was created as a control, amplified from the pK7WGF2 vector. Synthesis of double stranded RNA (dsRNA; primers in Supplementary Table 1) and nematode soaking were carried out as described in Chen et al. (2005). For each silencing construct tested, 10 three-week-old potato plants (cultivar Désirée) derived from internodal cuttings and cultured in compost:sand mixture (1:1) in root trainers in a glasshouse (16-h daylight, 22 ± 6°C) were inoculated with about 200 *G. pallida* J2s from the standard Pa2/3 population “Lindley” soaked in dsRNA derived from *GpSPRY-414-2* or *GFP*. The degree of infection was evaluated 3 weeks after inoculation by counting the number of females and early stage nematodes in roots stained with acid fuchsin under a stereo microscope. For this, roots were individually wrapped in Miracloth (Millipore), soaked in 1% bleach for 5 min, washed intensively with tap water and then boiled for 4 min in 60-times diluted acid fuchsin solution [0.35 g acid fuchsin, 25 ml glacial acetic acid, and 75 ml water], washed again with tap water, unwrapped and kept in destaining solution [glycerol containing 0.1% glacial acetic acid] until counting. Gene silencing in dsRNA-treated worms was checked by semi-quantitative reverse-transcription polymerase chain reaction (semi-qRT-PCR) using gene specific primers designed outside the region chosen for the silencing (Supplementary Table 1). The cDNA templates were prepared using the Superscript III kit (Life Technologies) from DNase-treated mRNA extracted using Micro Fast Track kit (Life Technologies) from the soaked or control nematodes, following the manufacturer's protocols. Each PCR reaction contained 1 μl of cDNA, 5 μl 10x GoTaq PCR buffer (Promega), 2 μl 10 mM dNTPs, 1.5 μl of each primer at 10 μM, 0.2 μl GoTaq DNA polymerase (Promega) and water to 50 μl. Cycling conditions consisted of one cycle of denaturing at 95°C for 2 min followed by 35 cycles of 95°C denaturing for 45 s, 53°C or 59°C annealing for 30 s (for *SPRYSEC* genes or *EF1*α control, respectively) and 72°C extension for 20 s. A final extension was done for 3 min at 72°C. PCR products were run on 1.5% agarose gels and then visualized by ethidium bromide staining.

### Yeast two-hybrid (Y2H) analysis

The ProQuest system (Life Technologies) was used for the Y2H screening. The cDNA library, made commercially from pathogen infected Désirée potato leaf material, was cloned in pDEST22 (Bos et al., 2010). Yeast Mav203 cells were co-transformed with 1 μl potato cDNA library (1 μg/μl) and 2 μl *GpSPRY-414-2* bait (50 ng/μl) cloned in pDEST32. Yeast transformants were plated out on synthetic Leu and Trp dropout media to evaluate the depth of the screen (over 2 million clones were screened) and on triple dropout media (Leu, Trp, His) for the identification of the candidate interactors. In the triple dropout media, 10 mM 3-Amino-1,2,4-triazole was added to suppress self-activation of the *HIS3* gene. Eleven colonies were recovered from the interaction selective plates that were picked and re-plated on triple dropout media (Supplementary Table 2). Freshly grown yeast colonies were then used to test interactions in the subsequent reporter gene assays (following the Y2H ProQuest Characterization of Transformants procedure). Candidate transformants were regarded as positive if they grew on the triple dropout media and turned blue in the X-gal assay. Each interacting prey candidate clone was then purified from yeast, rescued into *E. coli* DH5α and sequenced. Preys for which the sequence was not in frame with the GAL4 activation domain were discarded (3 out of 11). In order to confirm interactions for the selected prey clones, each unique identified potato prey clone was then co-transformed one-to-one with *GpSPRY-414-2* into Mav203. Similarly, three other *GpSPRYSEC* bait constructs were tested to assess the specificity of the interaction (*GpSPRY-24D4, GpSPRY-17I9-1*, and *GpSPRY-12N3*). From each transformation, at least two independent clones were selected that were tested with the same reporter gene assays as described above. In addition, prey and bait were individually tested for absence of auto-activation by co-transformation into Mav203 together with empty bait or prey vector.

### *In planta* localization and bimolecular fluorescence complementation (BiFC) assay

The subcellular localization of *Gp*SPRY-414-2 and its putative plant target *St*CLASP (as full length or truncated versions corresponding to the Y2H G1-5 insert fragments) was investigated using proteins fused to fluorescent tags for confocal analysis. *Agrobacterium*-mediated transient expression in *N. benthamiana* and imaging by confocal microscopy were performed as described in Thorpe et al. (2014). For co-localization analysis with subcellular tubulin marker GFP::TUA6 (Ueda et al., 1999), bacteria were infiltrated in leaves of transgenic *N. benthamiana* line (Gillespie et al., 2002). For BiFC analysis, the *YFPc::GpSPRY-414-2* construct in pCL113, as well as the negative control *YFPc::GpSPRY-24D4* in pCL113, and *YFPn::CLASP-Y2H* or *YFPn::StCLASP-FL* in pCL112 were used. Complementary split-YFP constructs in *A. tumefaciens* strain GV3101 were co-infiltrated in *N. benthamiana* leaves at OD_600nm_ 0.02 and 0.1 for the *SPRYSEC* and either of the *StCLASP* clones, respectively. Two days later, the YFP was imaged with an excitation wavelength (λ) of 514 nm and emission collected at λ530–575 nm on Zeiss LSM 710 confocal.

### Protein extraction and western blot analyses

For Western blot analyses, the same combinations of constructs used in BiFC assays were transiently expressed in *N. benthamiana* as described above, except that bacteria were inoculated at OD_600nm_ 0.2 and 0.4 for the *SPRYSEC* and either of the *StCLASP* clones, respectively. Samples were collected 3 days post inoculation (dpi). For each combination 6 patches of leaf tissue were sampled from 3 plants using a size 5 Cork-borer, pooled into a 2-ml Eppendorf tube and flash frozen in liquid nitrogen. One stainless steel bead (4–mm diameter) was added to the tube and the samples were disrupted for 40 s at a frequency of 30 Hz using a TissueLyserII (Qiagen). Each sample was prepared in duplicate for protein extraction in native and in denaturing conditions. For extraction, the tissue powder was homogenized in 0.6 ml of extraction buffer containing 10% glycerol, 25 mM Tris-Cl pH 7.5, 1 mM EthyleneDiamineTetraAcetic acid (EDTA), 150 mM NaCl, 2% (w/v) PolyVinylPolyPyrrolidone (PVPP), 1 mM PhenylMethylSulfonyl Fluoride (PMSF), and 2 μl/ml plant proteinase inhibitor cocktail (SIGMA P9599). For extraction in denaturing conditions, the buffer was supplemented with 10 mM DiThioThreitol (DTT) and 0.15% (v/v) Nonidet P-40. Samples were incubated on ice for 10 or 20 min for the native and denaturing protein extractions, respectively. The extracts were purified by centrifugation (10,000 g for 1 min at 4°C) and the supernatant collected in a fresh tube. This centrifugation step was repeated two more times and the final protein extracts were split into 75-μl aliquots.

For Western blotting, the protein samples were either mixed with loading buffer (Life Technologies BN2003), subjected to non-denaturing electrophoresis on NativePAGE Novex 3–12% Bis-Tris gels (Life Technologies) and subsequently transferred to PVDF membranes in an XCell-II blot module (Life Technologies), or heat denatured (5 min in boiling water) in loading buffer (Life Technologies NP0007), subjected to SDS-PAGE on NuPAGE Novex 4-12% Bis-Tris gels (Life Technologies) and subsequently transferred to Nitrocellulose membranes, following the manufacturer's instructions. Gels were run at 4°C for 2 h at 150 V and transferred for 2 h. Membranes were stained with Ponceau Red staining solution (SIGMA) followed by destaining in sterile distilled water prior to blocking in 5% (w/v) milk Phosphate Buffered Saline solution (PBS; 8 mM Na_2_HPO_4_, 1.5 mM KH_2_PO_4_, 2.7 mM KCl, 137 mM NaCl, pH 7.4) with Tween 20 at 0.1% (v/v) for 2 h. For the detection of the split-YFP fusions, the blots were incubated over-night at 4°C in 5% (w/v) milk PBST solution containing a primary antibody anti-GFP: either a polyclonal antibody (Santa Cruz sc8334 (Rabbit) used at 1:2,000) or a monoclonal antibody (Chromotek 3H9 (Rat) used at 1:2,000). Blots were rinsed 3 times for 10 min in PBST and then incubated at room temperature for 1 h in 5% milk PBST with a secondary antibody coupled to horseradish peroxidase (Goat anti-rabbit IgG SIGMA A0545 used 1:80,000 or Goat anti-rat IgG SIGMA A9037 used 1:10,000). Blots were rinsed 3 times for 10 min in PBST and then 5 min in PBS without Tween 20 prior to chemiluminescent detection using Pierce-Thermo Scientific SuperSignal West Pico and Femto substrates with CL-Xposure films (Thermo Scientific).

### flg22-mediated reactive oxygen species (ROS) production suppression assay

Free *eGFP* control (in pK7WG2; Mei et al., 2015) and *eGFP*::*GpSPRY-414-2* construct (in pK7WGF2) were transiently expressed in *N. benthamiana* leaves using the *Agrobacterium*-mediated expression system as for the localization experiments described above, except that bacteria suspended in infiltration buffer were incubated overnight in the dark at room temperature prior to further dilution in infiltration buffer. Next morning, bacteria were then spot-inoculated at OD_600nm_ 0.3. About 30 h post inoculation, leaf discs were sampled using a size 2 Cork-borer and floated on water overnight in 96-well plates (24 replicates per construct sampled from at least 8 different plants). Active oxygen species production was elicited with the bacterial PAMP flg22 peptide (synthetic peptide QRLSSGLRINSAKDDAAGLAIS; PeptideSynthetics, UK) and measured by a Luminol-dependent assay 48 h post inoculation. Briefly, water was replaced by a solution containing flg22 (100 nM), horseradish peroxidase (20 μg/ml HRP; SIGMA) and L-012 (0.5 mM Luminol derivative; Waco Chemicals, Germany). Luminescence was measured as relative luminescence units (RLU) using a plate-reader luminometer (SpectraMax-M5; Molecular Devices) over time (60-min kinetic with measures taken every second) with 750 ms integration time.

### Cell death suppression assay

A cell death suppression assay was performed with the cell death inducers Gpa2/RBP-1, Rx/PVX-CP, Cf-4/Avr-4, Cf-9/Avr-9, R3a/ Avr3a^KI^, R2/Avr2, autoactive Mi1.2^T557S^, and INF1, as described in Mei et al. (2015). These constructs were co-infiltrated in *N. benthamiana* leaves with either *eGFP* as a control or *eGFP::GpSPRY-414-2* (in pK7WGF2).

### Microtubule network disruption using drug treatment

A solution of 100 μM colchicine was co-infiltrated with the *Agrobacteria* in leaf tissues to disrupt the microtubule network and the effects of this treatment on BiFC analysis as well as on ROS production and the cell death suppression assays were examined. These assays and confocal imaging were performed as described above. The microtubule network was still disrupted 5 days after the colchicine treatment (data not shown) but to maintain the effects of the drug, a second application of colchicine was carried out at 4 dpi during the cell death suppression assay.

## Results

### *Gp*SPRY-414-2 is a SPRYSEC effector

Our previous work (Mei et al., 2015) allowed the identification of a substantial *SPRYSEC* gene family in *G. pallida*. One of these, *GpSPRY-414-2*, was cloned from cDNA of J2 nematodes. *GpSPRY-414-2* without signal peptide encodes a 210-amino acid protein with a predicted molecular mass of 23.2 KDa. It has one SPRY domain, predicted by SMART analysis from amino acid 65 to 196 (Supplementary Figure S1). An *in situ* hybridization assay demonstrated that *GpSPRY-414-2* is expressed specifically in the dorsal pharyngeal gland cell of *G. pallida* J2s, indicating that it most likely encodes a secreted protein which may have a role in plant parasitism (Mei et al., 2015).

**Figure 1:**
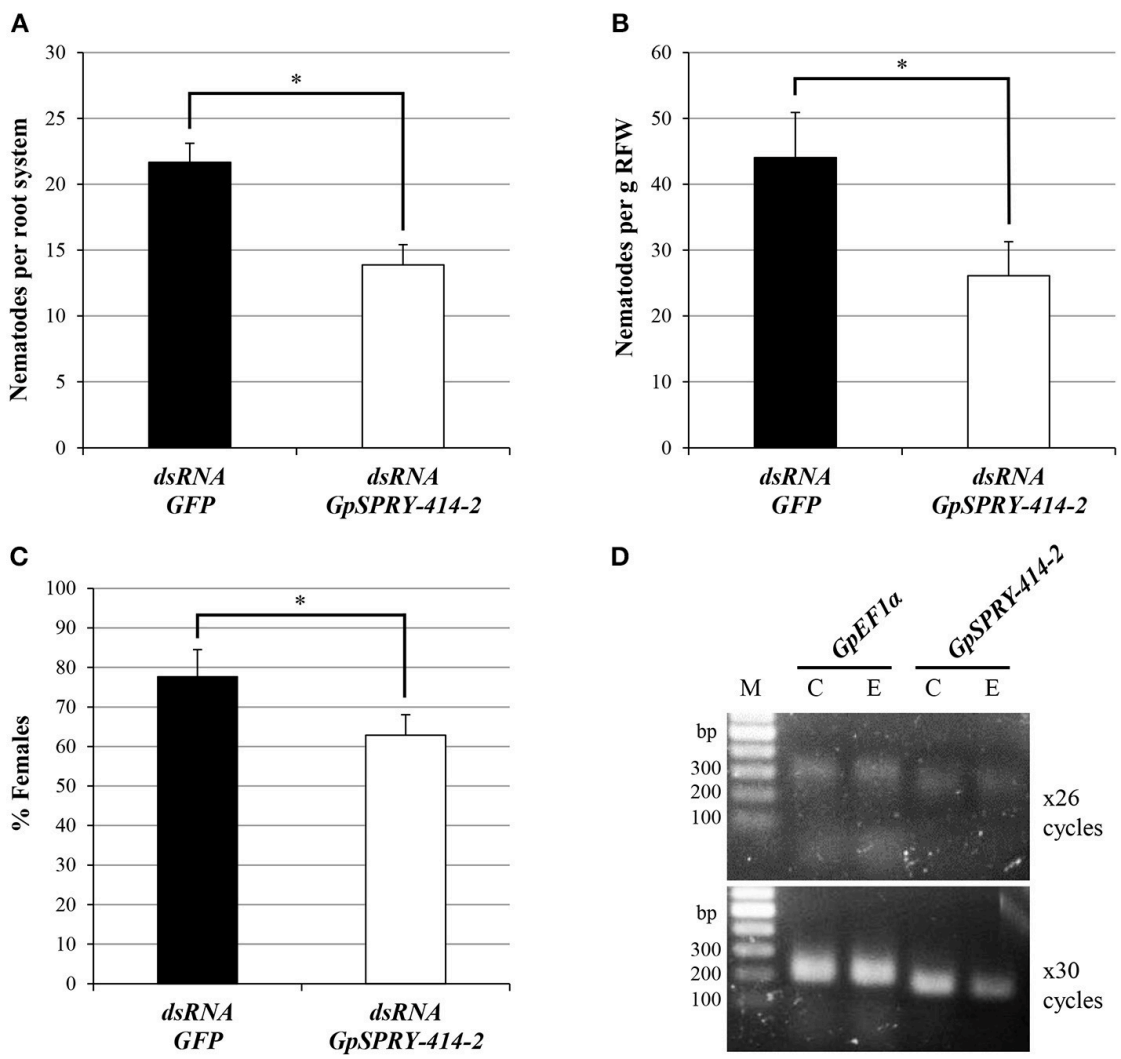
Silencing of the nematode effector gene *GpSPRY-414-2* reduces parasitic success of *G. pallida* in potato. **(A–C)** Nematode parasitism evaluated 3 weeks after inoculation by preparasitic J2s exposed to dsRNA from *eGFP* (control in black) or *GpSPRY-414-2* (in white) and expressed as **(A)** total number of nematodes per plant or **(B)** per gram of root fresh weight (RFW) and **(C)** percentage of females. Bars represent mean ± SE of 10 replicates with significant difference of means (Student's *t*-test at *P* < 0.05) indicated by an asterisk. This silencing experiment has been performed twice. **(D)** Semi-quantitative reverse-transcription polymerase chain reaction for *GpSPRY-414-2* and the *Elongation Factor 1 alpha* (*GpEF1*α) transcripts in control nematodes soaked in *eGFP* dsRNA [C] and nematodes exposed to *GpSPRY-414-2* effector dsRNA [E]. Reactions were sampled after 26 and 30 cycles. M = DNA ladder.

RNA interference-mediated gene silencing of the *G. pallida GpSPRY-414-2* significantly reduced infectivity of the pathogen (Figure 1). On average, the number of nematodes found in potato roots 3 weeks after inoculation was decreased by 40% in the *GpSPRY-414-2* dsRNA soaked samples when compared with a dsRNA *GFP* control (Figures 1A,B). In addition, a significant reduction in the percentage of females was observed (Figure 1C) in *GpSPRY-414-2* silenced samples. The gene silencing in nematodes prior infection was confirmed by semi-qRT-PCR, using *GpEF1*α as constitutive control (Figure 1D). The significant impact of the partial gene silencing on infection indicates that *GpSPRY-414-2* is important for parasitism.

### *GpSPRY-414-2* suppresses plant defense responses

**Figure 2:**
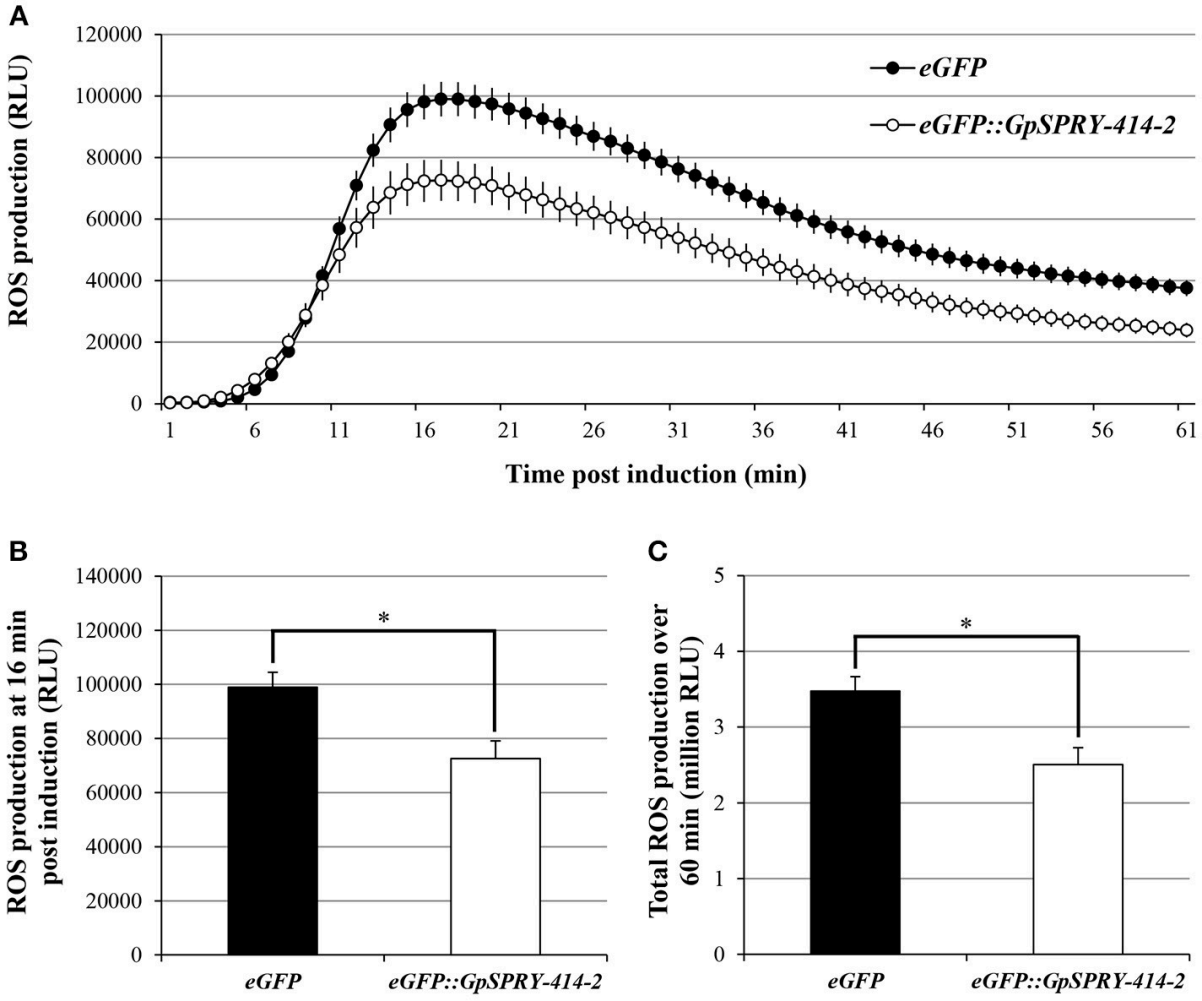
*Gp*SPRY-GPE414-2 suppresses flg22-induced ROS production in *N. benthamiana*. Reactive oxygen species (ROS) production induced by flg22 (100 nM) in *N. benthamiana* leaves expressing *eGFP* (black) or *eGFP::GpSPRY-414-2* (white). ROS production is shown as time-course after elicitation by flg22 in panel **(A)**, at peak of ROS production (16 min post-elicitation) in panel **(B)** or expressed as total relative light units (RLU) over 60 min following elicitation in panel **(C)**. Values indicated are average RLU ± SE of 24 leaf disc samples with significant difference of means (Student's *t*-test at *P* < 0.05) indicated by an asterisk in panels **(B)** and **(C)**. This experiment was repeated three times in total. Representative control samples for the ROS assay are presented in Supplemental Figure S5.

A burst of reactive oxygen species is one of the earliest responses usually associated with the induction of PTI and can be triggered by the bacterial-derived PAMP flg22 peptide. Transient expression of *eGFP::GpSPRY-414-2* in *N. benthamiana* suppressed flg22-induced ROS production in leaf explants when compared with the *eGFP* control (Figure 2).

**Figure 3:**
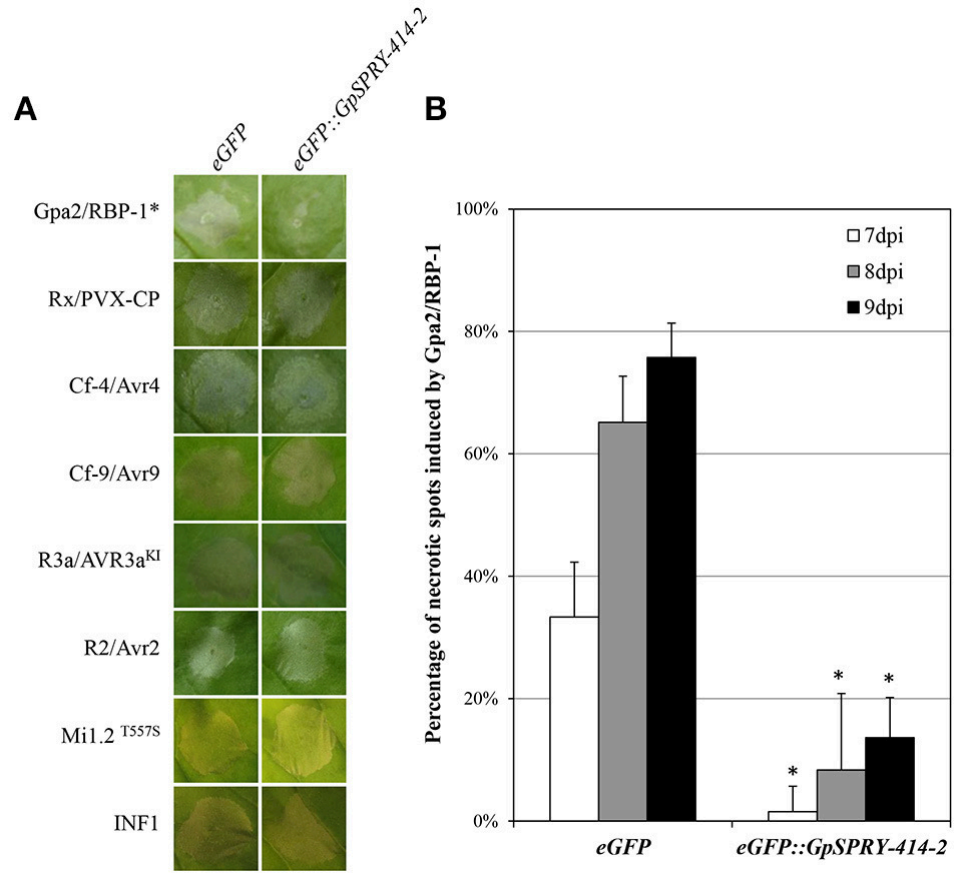
*Gp*SPRY-414-2 specifically suppresses plant programmed cell death mediated by Gpa2 and RBP-1. **(A)** Cell death symptoms induced in *N. benthamiana* by co-expression of *R2/Avr2* [3 days post infiltration (dpi)], *R3a/Avr3a*^*KI*^ (2 dpi), *Cf-4/Avr4* (4 dpi), *Cf-9/Avr9* (4 dpi) *Rx/PVX-CP* (3 dpi)*, Gpa2/RBP-1* (7 dpi), an autoactive form of Mi-1.2 (Mi-1.2^T557S^; 3 dpi), or the *P. infestans* PAMP elicitor *INF1* (3 dpi) were recorded in leaves expressing either the free enhanced green fluorescent protein (eGFP) as a control or the eGFP-tagged *Gp*SPRY-414-2 fusion. The asterisk indicates the combination where the symptoms are significantly suppressed by the effector compared to eGFP. Typical symptoms presented are representative of 10–12 plants infiltrated on two leaves per combination. The overall set of infiltration was performed at least twice for each cell death inducer. **(B)** Percentage of infiltration sites developing a clear hypersensitive response (HR) from 7 to 9 dpi mediated by Gpa2/RBP-1 in *N. benthamiana* leaves expressing the free *eGFP* or the *eGFP::GpSPRY-414-2* fusion. The experiment was replicated three times with blocks of 11 plants infiltrated on two leaves each and both constructs tested on each leaf. The results presented are the combined data for the 66 infiltration spots. Values represent means ± SE (*n* = 3) with significant difference (Mann-Whitey U test at *P* < 0.05) indicated by an asterisk for *eGFP::GpSPRY-414-2* as compared to the free *eGFP* control gene evaluated at the same time point.

The ability of *Gp*SPRY-414-2 to suppress ETI was investigated using a range of cell death inducers, including many resistance and avirulence gene pairs. However, transient expression of *eGFP::GpSPRY-414-2* in *N. benthamiana* only suppressed ETI mediated by Gpa2 and RBP-1 (Figure 3A). Analysis from large-scale infiltrations confirmed the significant reduction in percentage of necrotic spots for *eGFP::GpSPRY-414-2* treatment compared to the *eGFP* control for up-to 9 dpi (Figure 3B).

### *Gp*SPRY-414-2 interacts with a potato clasp in yeast

**Figure 4:**
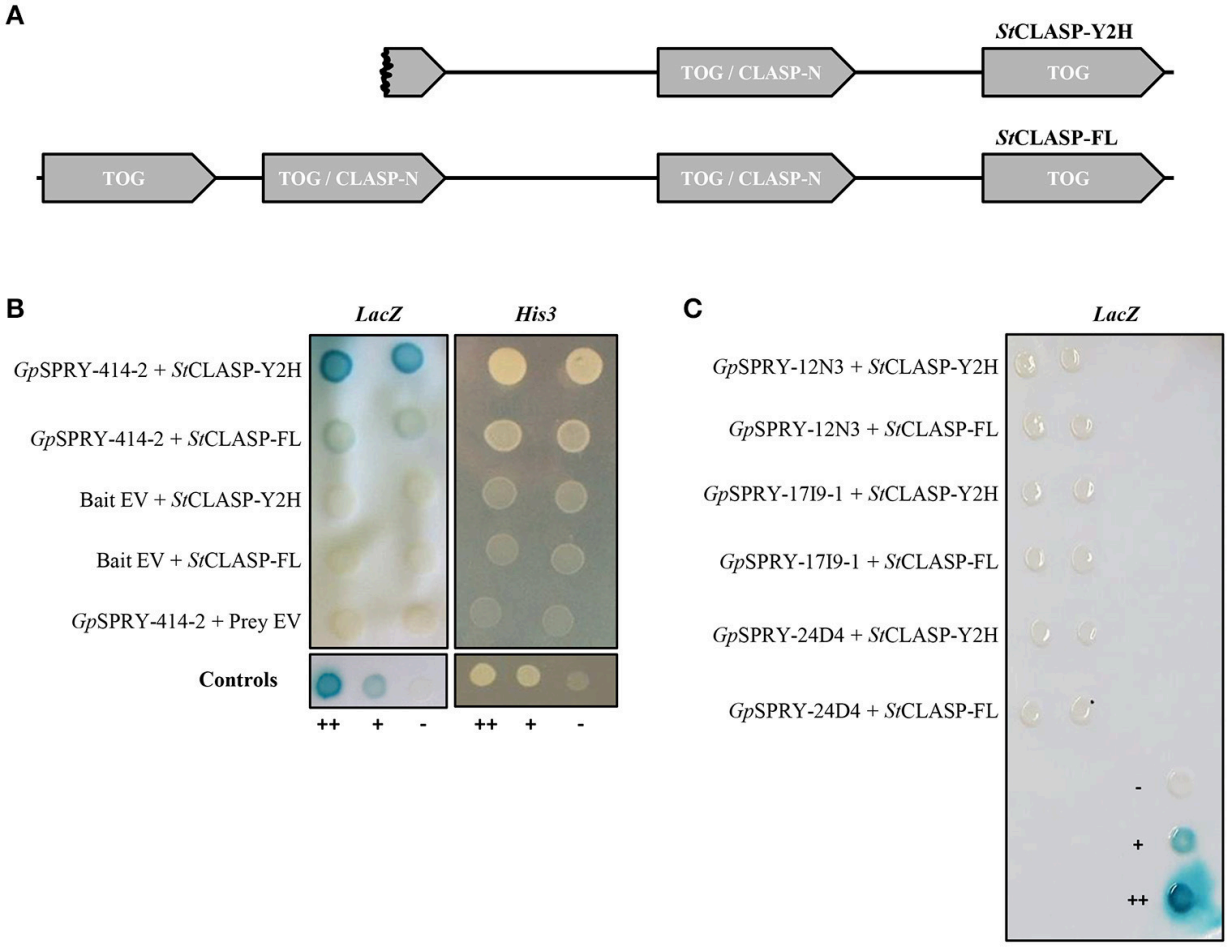
Yeast two-hybrid interactions between *Gp*SPRY-414-2 and the potato CLASP. **(A)** Schematic diagram showing the domain architecture of the truncated (partial protein clone G1-5; *St*CLASP-Y2H) and full-length CLASP protein from potato (*St*CLASP-FL), containing TOG domains (Tubulin-binding tumor Overexpressed Gene) and CLASP-N regions according to SMART analysis. **(B)** *Gp*SPRY-414-2 interacts with the full-length and truncated CLASP proteins, according to the activity of both *His3* and *LacZ* reporter genes in the yeast two-hybrid (Y2H) screening. The empty vectors pDEST32 (bait) and pDEST22 (prey) were used as control to test the proteins for absence of auto-activation in the Y2H system. **(C)** Full length *St*CLASP and truncated form do not interact with other SPRYSECs in yeast. Yeast colonies are presented in duplicates that correspond to independent clone transformants. ++ control yeast from the Life Technologies ProQuest™ Two-Hybrid System for strong interaction, + for weak interaction and – for no interaction. Reporter assays were repeated at least twice for each combination.

A Y2H screening was performed against a potato cDNA prey library to identify potential host interactors of *Gp*SPRY-414-2 (Supplementary Table 2). This screening revealed that the *Gp*SPRY-414-2 could interact with a putative CLASP protein. From the original screening, 5 prey clones out of 11 were recovered with sequences related to CLASP, which included 3 unique partial sequences with different 5′ end. The clone G1-5, which had the longest 5′ end and consisted of a partial CLASP coding sequence later referred to as *StCLASP-Y2H*, was the only clone confirmed as interactor after one-to-one transformation with the *GpSPRY-414-2* bait construct and testing for lack of auto-activation of the reporters by either of the proteins (Figure 4). The full length *CLASP* coding sequence (later referred to as *StCLASP-FL*) was therefore cloned from potato.

A BLASTn search, with the *StCLASP-Y2H* sequence including the 3′ UTR, against the potato genome (DM v3 superscaffolds dataset), identified two superscaffolds containing potential matches: PGSC0003DMB000000504 (chromosome 6) and PGSC0003DMB000000115 (chromosome 9) with respectively 88 and 99% sequence identity on average across all genomic fragments aligned. However, gene loci were not annotated at these locations at the time of the screening. Therefore, the closest annotated organism to potato, tomato, was used to help predict the gene structure and design primers for the cloning. A tBLASTn search with the *St*CLASP-Y2H sequence in the tomato genome identified two gene loci with 97% (Solyc09g063030) and 80% (Solyc06g008040) amino acid identity respectively. The sequences of the tomato gene locus Solyc09g063030 and the potato candidate gene from superscaffold PGSC0003DMB000000115 were reciprocal best blast hits and the closest homologous sequences to *StCLASP-Y2H* (Supplementary Figure S2).

The *StCLASP-FL* (4,290 bp) that was cloned from the potato cultivar Désirée is identical to the GenBank accession XM_006350231.2. It corresponds to the protein XP_006350293.1, which is predicted as a CLIP-associated protein-like in cultivar DM 1-3 516 R44. The *St*CLASP-FL protein (1,429 amino acids) has 81% identity with the other potato *St*CLASP candidate (XP_015159473.1) as well as 97 and 81% identity with the two tomato *Sl*CLASPs, Solyc09g063030 and Solyc06g008040, respectively. All these proteins have a similar architecture (Supplementary Figure S2) with 4 TOG domains that are found in Tubulin-binding tumor Overexpressed Gene (InterPro domain IPR034085). It was recently proposed that TOG proteins have distinct architectures and tubulin-binding properties that underlie each family's ability to promote or pause microtubule polymerization (Byrnes and Slep, 2017). The two middle TOG domains of the Solanaceous CLASP considered here above can also be predicted with high confidence as CLASP-N domains (InterPro domain IPR024395). The CLASP_N region is found at the N-terminal end of CLIP-associated proteins (CLASPs), which are widely conserved microtubule plus-end tracking proteins that regulate the stability of dynamic microtubules (Galjart, 2005). The partial *St*CLASP-Y2H covers more than 2/3 of the full length *St*CLASP from the C-terminus, including the second CLASP-N domain and a small part of the first CLASP-N domain (Figure 4A). Both of these proteins interacted in yeast with the SPRYSEC bait *Gp*SPRY-414-2, although the interaction with *St*CLASP-FL protein was weaker than with the original partial sequence *St*CLASP-Y2H (Figure 4B). Neither full-length nor partial *St*CLASPs could interact with three other SPRYSECs tested (*Gp*SPRY-24D4, *Gp*SPRY-12N3, and *Gp*SPRY-17I9-1), indicating that the interaction with *Gp*SPRY-414-2 was most likely specific (Figure 4C).

### Interaction of *Gp*SPRY-414-2 with the potato CLASP *in planta*

**Figure 5:**
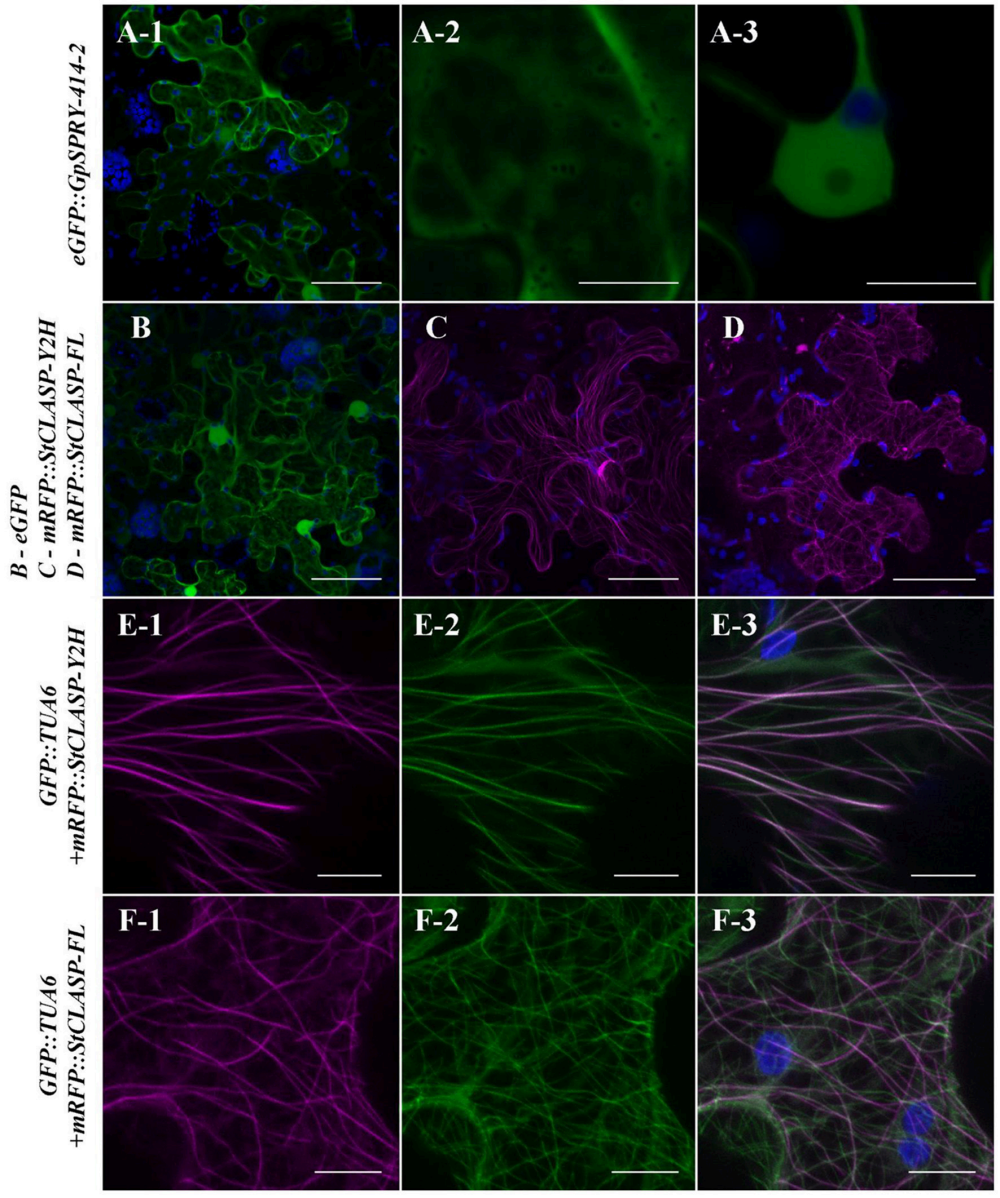
*In planta* localization of *Gp*SPRY-414-2 and its putative plant target *St*CLASP. *Agrobacterium*-mediated transient expression of the proteins tagged N-terminally with fluorescent markers in *Nicotiana benthamiana* leaves. **(A)** *Gp*SPRY-414-2 fused to eGFP. Subpanels A-1 and A-2 represent maximum intensity projection images at the cellular level and focused on the cytoplasm, respectively. The subpanel A-3 is an image of a single section taken through the nucleus. **(B)** Free eGFP. **(C,D)** *St*CLASP proteins fused to mRFP with **(C)** *St*CLASP-Y2H representing the truncated form of the protein identified in yeast-two hybrid screen and **(D)** the full-length *St*CLASP-FL. **(E,F)** Truncated **(E)** and full-length **(F)** *St*CLASP proteins fused to mRFP and co-expressed in the transgenic *N. benthamiana* line expressing a GFP-tagged tubulin marker (TUA6). Images are maximum intensity projections with RFP presented in sub-panel 1, GFP in sub-panel 2 and an overlaid image in sub-panel 3. Pictures were all taken 2 days post infiltration by confocal microscopy. The GFP and RFP signals are displayed in green and magenta, respectively. Autofluorescence from chloroplasts is displayed in blue. Scale bars in **(A-1,B–D)** represent 50 μm and in **(A-2,A-3,E,F)** represent 10 μm. Each localization experiment was replicated at least twice.

**Figure 6:**
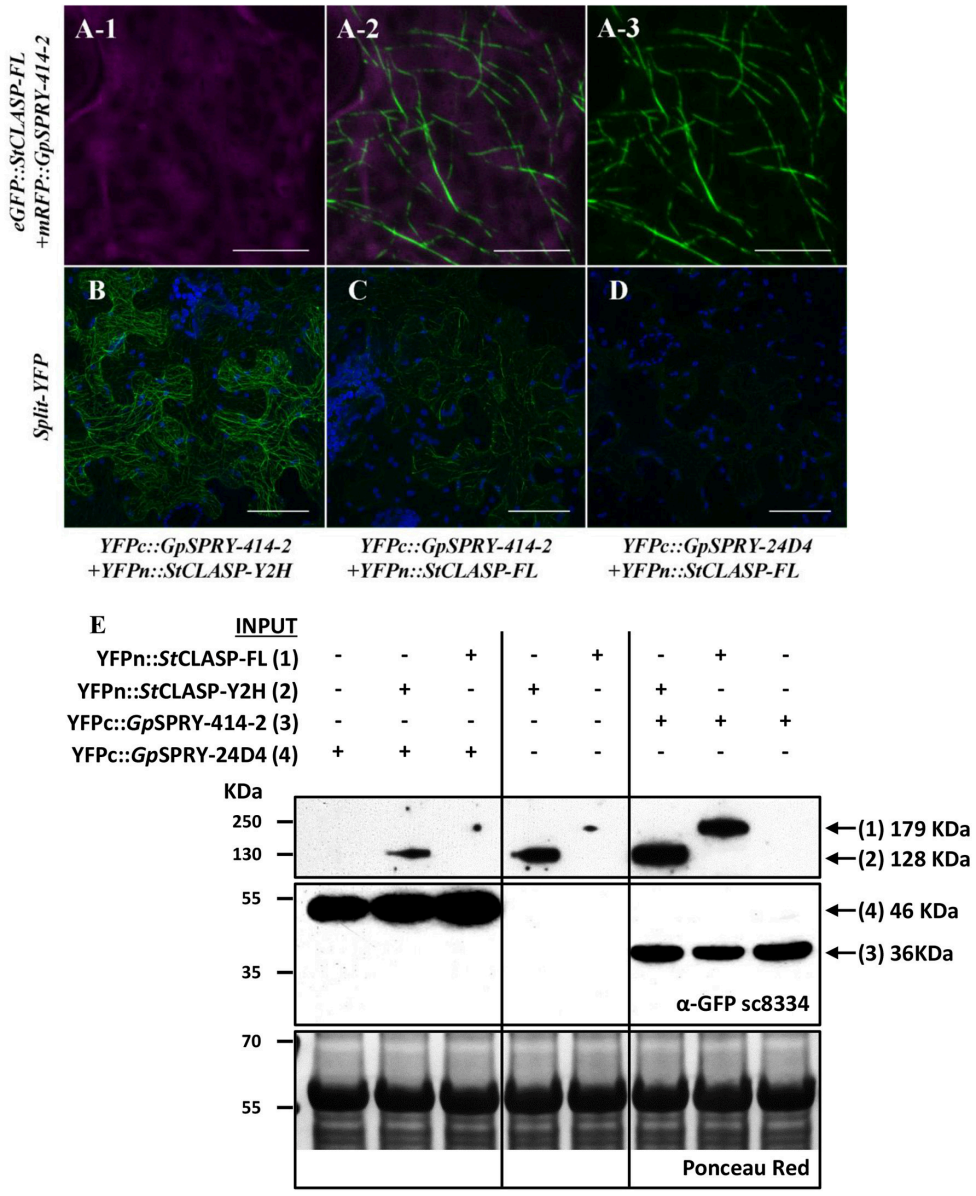
*Gp*SPRY-414-2 and *St*CLASP are cytoplasmic proteins that co-localize at the microtubules. **(A)** Localization of eGFP::*Gp*SPRY-414-2 **(A-1)** and mRFP::*St*CLASP-FL full-length protein **(A-3)** co-expressed transiently in *N. benthamiana* leaves. GFP and RFP signals displayed in green and magenta, respectively, with overlay presented in sub-panel **(A-2)**. Chloroplasts are displayed in blue. **(B–D)** Bimolecular fluorescence complementation (BiFC) assay using transient expression in *N. benthamiana*. Both truncated (**B**; YFPn::*St*CLASP-Y2H) and full-length *St*CLASP (**C**; YFPn::*St*CLASP-FL) may interact with YFPc::*Gp*SPRY-414-2 at the microtubules but generate different signal intensity in BiFC when observed under the same confocal imaging conditions, while very weak signal can be detected in presence of YFPn::*St*CLASP-FL and another SPRYSEC protein (YFPc::*Gp*SPRY-24D4) **(D)**. Reconstituted YFP is displayed in green and autofluorescence from chloroplasts is displayed in blue. Each experiment was done at least twice with three replicates. Scale bars represent 10 μm in A and 50 μm in **(B–D)**. **(E)** Immuno-detection by Western blot of the YFPn::*St*CLASP and YFPc-effector fusion proteins using the polyclonal antibody anti-GFP (α-GFP sc8334). A long exposure of the blot was required to detect the YFPn::*St*CLASP proteins and thus the framed images represent sections of the same blot taken after more or less time. The full blot after long exposure is presented as Supplemental Figure S4. The total amount of protein loaded per sample is represented in the Ponceau red panel for a section of the blot. The predicted size of each fusion protein is indicated on the right-hand side of the panel.

To investigate whether the interaction between *Gp*SPRY-414-2 and *St*CLASP could occur within plant cells, we examined their localization *in planta*. Fluorescent protein fusion constructs for *GpSPRY-414-2* and its putative target *StCLASP* (partial and full-length clones) were transiently expressed in *N. benthamiana* leaves. The N-terminally tagged effector *Gp*SPRY-414-2 is localized in the cytoplasm and nucleoplasm (Figure 5A). This pattern of localization was consistent, irrespective of the position of the tag in the fusion, and similar to the localization of the free eGFP (Supplementary Figures S3A,B, F–H). Both partial and full-length *St*CLASPs, also tagged at the N-terminus, were localized in the cytoplasm where, unlike free eGFP, they form structures similar to cytoskeletal filaments (Figures 5B–D). Co-expression with the TUA6 tubulin marker indicated that the *St*CLASPs co-localized with microtubules (Figures 5E,F), which is in agreement with the localization reported for the Arabidopsis characterized CLASP (Ambrose et al., 2007). The full length *St*CLASP showed the same localization pattern regardless of the position of the tag (Supplementary Figure 3E). The localization patterns of *Gp*SPRY-414-2 and *St*CLASPs remained the same when the proteins were concomitantly expressed, as observed for the full-length *St*CLASP on Figure 6A. Furthermore, we never observed any obvious changes in the microtubule network when either or both proteins (mRFP-tagged) were co-expressed with GFP::TUA6 (data not shown).

**Figure 7:**
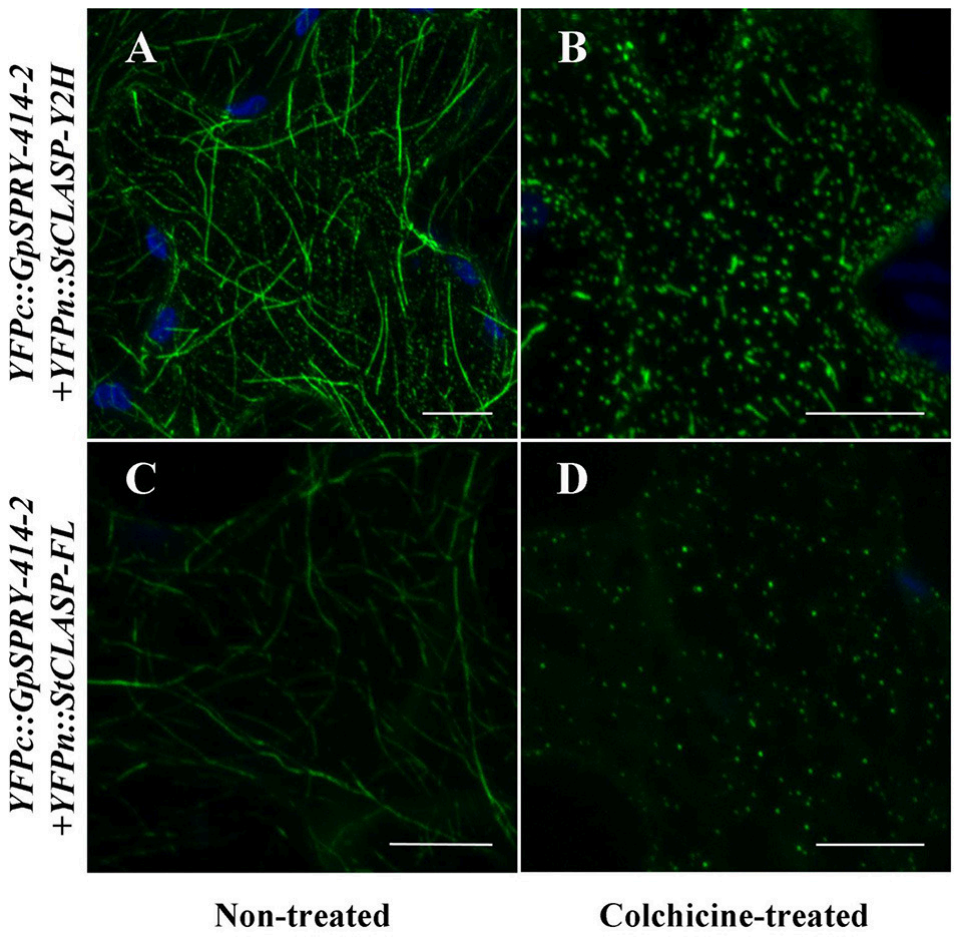
*Gp*SRPY-414-2 and *St*CLASP remain in close vicinity despite the disruption of the microtubule network by the colchicine treatment. Either partial (**A,B**; *YFPn::StCLASP-Y2H*) or full-length *StCLASP* (**C,D**; *YFPn::StCLASP-FL*) YFPn-fusion protein constructs were transiently expressed in *Nicotiana benthamiana* leaves with *Globodera pallida* YFPc-effector complementary construct (*YFPc::GpSPRY-414-2*) for co-localization by bimolecular fluorescence complementation (BiFC) assay, using plants treated **(B,D)** or not **(A,C)** with colchicine at 100 μM. Reconstituted YFP is displayed in green and autofluorescence from chloroplasts is displayed in blue. The experiment was repeated with at least two replicates per combination and per treatment. Scale bars represent 50 μm.

Further localization studies using BiFC assays showed that *Gp*SPRY-414-2 and either of the *St*CLASP constructs mainly co-localized at the microtubules (Figures 6B,C) and most probably interact with each other at this location as they are close enough to allow reconstitution of the split-YFP protein. The interaction between *Gp*SPRY-414-2 and *St*CLASP (either full-length or partial protein) was not affected by the microtubule depolymerizing drug colchicine (Figure 7). In the absence of colchicine, *Gp*SPRY-414-2 co-localized with *St*CLASPs on microtubule strings, while in the presence of colchicine, when the microtubule network was disturbed, the YFP signal was still observed but it was dispersed within the cytoplasm like microtubule fragments. In addition, in BiFC experiments a stronger YFP signal was consistently observed when *GpSPRY-*414-2 was expressed with the partial *St*CLASP. A similar pattern was observed in yeast where the strength of interaction was stronger between *Gp*SPRY-414-2 and *St*CLASP-Y2H than *St*CLASP-FL (Figure 4B, *LacZ* reporter assay). To confirm that the potential interaction of *Gp*SPRY-414-2 with *St*CLASP-FL observed by BiFC was genuine, the SPRYSEC candidate *Gp*SPRY-24D4 that did not interact with *St*CLASPs in yeast was also tested. Like *Gp*SPRY-414-2, *Gp*SPRY-24D4 is a cytoplasmic protein but it is mainly excluded from the nucleus (Supplementary Figures S3C,D). In a BiFC assay with *St*CLASP-FL, *Gp*SPRY-24D4 only showed a very weak and inconsistent signal despite good expression of the effector itself, as shown by Western blotting (Figures 6D,E).

**Figure 8:**
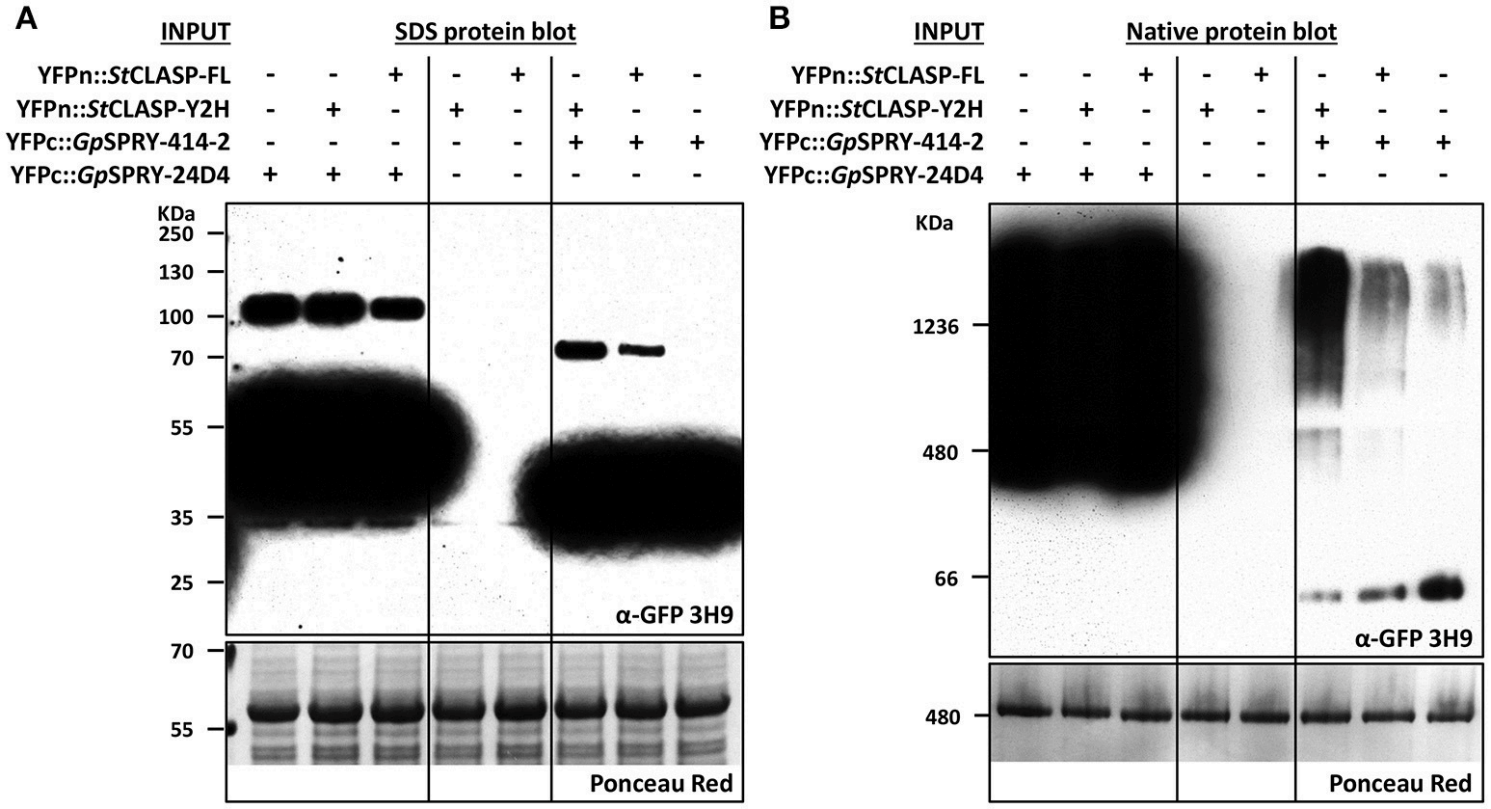
*Gp*SPRY-414-2 dimerizes and forms large protein complexes *in planta* in presence of *St*CLASP. Immuno-detection by Western blot in denaturing **(A)** or native conditions **(B)** of the split-YFP-tagged proteins used in bimolecular fluorescence complementation (BiFC) assay. *Globodera pallida* candidate effector genes *GpSPRY-414-2* and *GpSPRY-24D4* were transiently expressed in *Nicotiana benthamiana* leaves either alone or in combination with the *StCLASP* protein constructs (full length *StCLASP-FL* or truncated *StCLASP-Y2H*). The YFPc-effector fusion proteins were specifically detected using the monoclonal antibody anti-GFP (α-GFP 3H9). The *YFPn-StCLASP* constructs (not detected by this antibody) were also expressed alone as control. The total amount of protein loaded per sample is represented in the Ponceau red panels for a section of the blots. The predicted sizes for the SPRYSEC monomers (YFPc-fusions in denaturing conditions) are 36 and 46 KDa for *Gp*SPRY-414-2 and *Gp*SPRY-24D4, respectively.

The immuno-detection by Western blot (Figure 6E) of the split-YFP YFPn-*St*CLASP and YFPc-effector fusion proteins indicated, on the one hand, that both effector constructs were stably expressed despite the low or lack of YFP signal in BiFC experiments. On the other hand, it consistently demonstrated that the *St*CLASP proteins were rather unstable or recycled very fast as they were barely detectable on their own. However, in the presence of *Gp*SPRY-414-2, both partial and full-length *St*CLASP were stabilized. This was a specific effect that was most likely dependent on *Gp*SPRY-414-2 interaction with the *St*CLASP proteins as it never occurred in the presence of *Gp*SPRY-24D4 (Figures 6D,E). In addition, when the anti-GFP monoclonal antibody was used, which detects the C-terminal part of the YFP, we noticed the presence of bands corresponding to double the size of the SPRYSEC effector fusion proteins (Figure 8A). This interaction between the two SPRYSEC proteins was strong enough to allow some homodimers to persist in denaturing conditions. The formation of high molecular weight protein complexes including the SPRYSEC effectors was confirmed in non-denaturing conditions (Figure 8B). The homo-dimerization of the *Gp*SPRY-24D4 happened irrespective of the presence of *St*CLASP proteins. By contrast, the homo-dimerization of *Gp*SPRY-414-2 observed in denaturing conditions and the formation of a high molecular weight protein complex observed in native conditions were prompted by the presence of the *St*CLASP proteins. Moreover, the more stable the *St*CLASP protein appeared to be, the greater proportion of the *Gp*SRY-414-2 formed complexes. Altogether, the co-localization and protein stability studies strongly suggest that *Gp*SPRY-414-2 interacts with its *St*CLASP target *in planta*.

### The hypersensitive reaction mediated by Gpa2/RBP-1 and its suppression by *G*pSPRY-414-2 do not require a functional microtubule network

**Figure 9:**
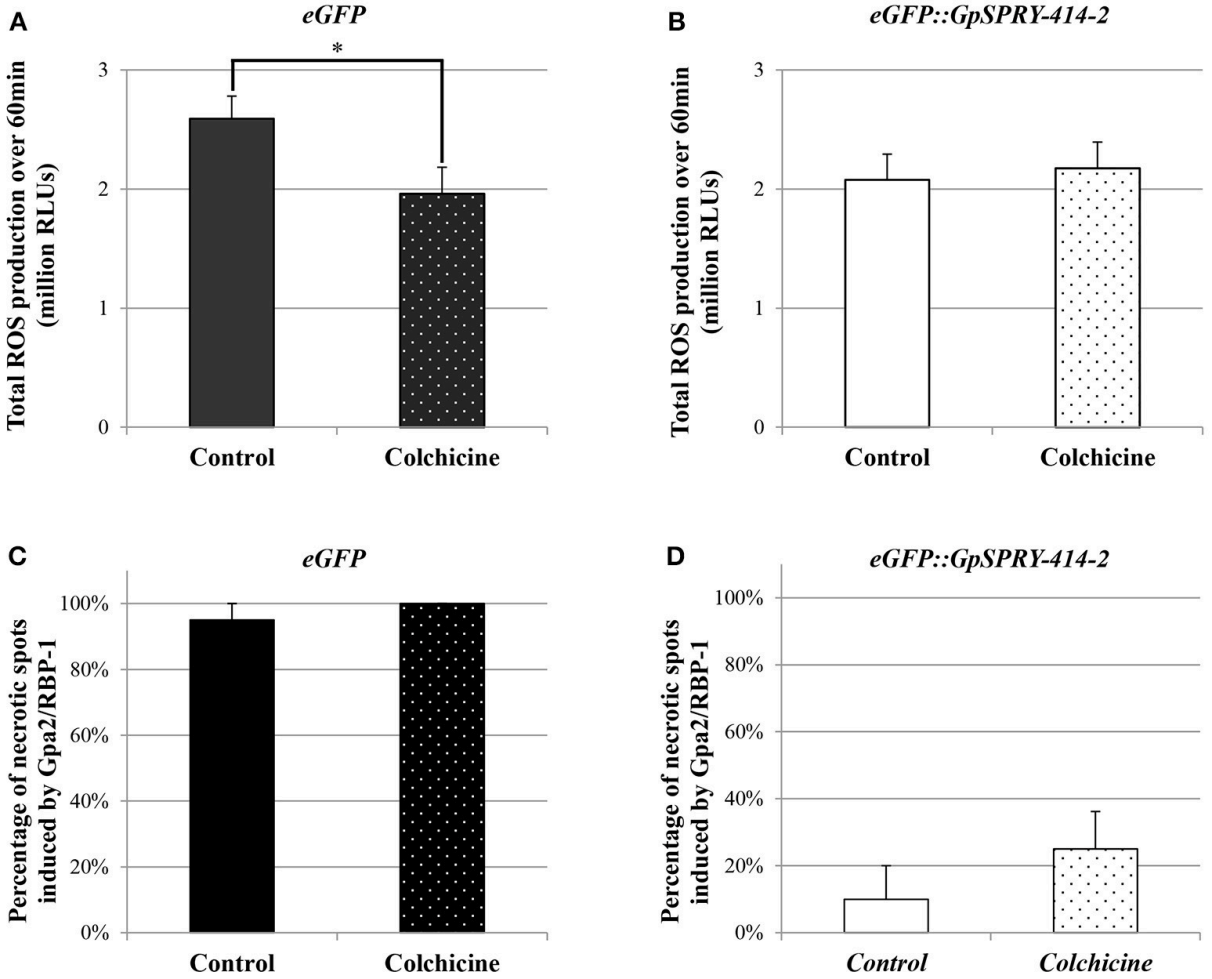
Disruption of the microtubule network in *N. benthamiana* using colchicine affects flg22-induced ROS production but does not impair Gpa2/RBP-1 mediated cell death or its suppression by *Gp*SPRY-414-2. **(A,B)** Reactive oxygen species (ROS) production induced by flg22 (100 nM) in *N. benthamiana* leaves expressing *eGFP* (A) or *eGFP::GpSPRY-414-2* (B) and treated (dotted pattern) or not (plain bars) with colchicine (100 μM). ROS production is expressed as total relative light units (RLU) over 60 min following elicitation with flg22. Values are mean ± SE of 24 leaf disc samples with significant difference (Student's *t*-test at *P* < 0.05) indicated by an asterisk. This experiment was done twice. **(C,D)** Cell death symptoms induced in *N. benthamiana* leaves by co-expression of *Gpa2/RBP-1* in presence of *eGFP* (C) or *eGFP::GpSPRY-414-2* (D) were scored at 7 dpi in leaves that were treated (dotted pattern) or not (plain bars) with colchicine (100 μM). Experiments were done twice, each with no less than 10 plants per treatment (+/− colchicine) that were spot infiltrated on two leaves with all the construct combinations per leaf. Values represent means ± SE (*n* = 10) for one experiment, with no significant differences between control and colchicine-treated plants (Student's *t*-test at *P* < 0.05).

Since *St*CLASP is involved in microtubule organization in plant cells, we tested whether disrupting the microtubule network could affect the plant defense suppression effects mediated by *Gp*SPRY-414-2. For this, the microtubule-depolymerizing agent colchicine was co-infiltrated with the *Agrobacteria* during the cell death assay. The results showed that colchicine did not affect the signaling triggered by Gpa2 and RBP-1 as the percentages of necrotic spots for both treated and untreated eGFP samples were not significantly different (Figure 9A). Moreover, *Gp*SPRY-414-2 suppressed the cell death induced by Gpa2 and RBP-1 by more than 70%, irrespective of the colchicine treatment (Figure 9B). In conclusion, a functional microtubule network is not required for either the hypersensitive response provoked by Gpa2 and RBP-1 or the suppression of this defense response by *Gp*SPRY-414-2.

### ROS production induced by flg22 appears partly dependent on the integrity of the microtubule network

The suppression of flg22-induced ROS production mediated by *Gp*SPRY-414-2 was also tested in presence of colchicine. The microtubule-depolymerizing drug was infiltrated in *N. benthamiana* leaf tissues together with the *Agrobacteria* mediating transient expression of the eGFP-tagged effector or the free eGFP. The results showed that colchicine significantly affected ROS production induced by flg22 in presence of eGFP (Figure 9C). The colchicine on its own decreased by 15–25% the total ROS produced, which is similar to the effect of *Gp*SPRY-414-2 observed previously in the absence of drug (Figures 2, 9D). However, no further suppression was observed when *Gp*SPRY-414-2 was expressed in combination with the drug (Figure 9D).

## Discussion

### *Gp*SPRY-414-2 is an effector protein contributing to successful parasitism of *G. pallida*

*Globodera pallida* delivers effectors into host tissues to sustain its biotrophic life style. The SPRYSECs are a substantial gene family from this nematode and there is accumulating evidence that shows their importance in plant–cyst nematode interactions (Sacco et al., 2009; Postma et al., 2012; Mei et al., 2015). In this study, we focused on one new member, *Gp*SPRY-414-2, and explored its functions. The gene encoding the *Gp*SPRY-414-2 protein is expressed specifically in the dorsal gland cell of J2 nematodes and this SPRYSEC can suppress plant defenses. Our data indicate that this protein can not only suppress PTI by reducing flg22-induced ROS production but also specifically suppresses ETI triggered by the potato *R* gene *Gpa2* and its cognate nematode avirulence factor *RBP-1*. Suppression of ETI does not reflect the virulence function of all SPRYSECs but is a characteristic shared by several members of this effector family. Like *Gp*SPRY-414-2, they selectively suppress immunity-associated cell death and therefore are not general plant cell death suppressors (Diaz-Granados et al., 2016). To further test the function of *Gp*SPRY-414-2 in nematode infection, we silenced the effector in *G. pallida* using RNAi. The infection assay using juveniles soaked in dsRNA showed a significant decrease in *G. pallida*'s capability to colonize host plants with a reduced total number of nematodes and percentage of females in roots compared to control treatment. The reduction in the proportion of female nematodes is significant as sex is environmentally determined in *G. pallida;* nematodes that are able to obtain a good supply of nutrients become female while those that have a more restricted food intake become male (Trudgill, 1967). These data therefore suggest that a reduction in *Gp*SPRY414-2 levels impacts on the ability of the nematode to induce a fully functional feeding site. Taken together, these data indicate that *Gp*SPRY-414-2 is involved in parasitism.

### The *Gp*SPRY-414-2 effector targets a potato microtubule-associated CLASP

To get insights into the biological function of *Gp*SPRY-414-2, we searched for potential host targets. Using a combination of Y2H and *in planta* assays, we showed a specific association of the nematode *Gp*SPRY-414-2 effector with *St*CLASP, a CLIP-associated protein from potato involved in the regulation of the dynamics of microtubules. CLIPs and CLASPs target the plus-end of microtubules and thus are called +TIPs, for “plus-end tracking proteins” (Galjart, 2005). Transient expression of the full-length potato *StCLASP* in *N. benthamiana* did not reveal a plus-end localization of the protein but showed labeling of the total microtubule. This may be due to the high expression levels that are generated in the experimental system used here and is in agreement with the report of Ambrose et al. (2007), who showed that plus-end tracking could only be observed at low transgene expression levels; this is also consistent with reports from animal CLASPs and other +TIPs.

Further investigations were carried out in order to determine whether CLASP is involved in the suppression of plant defenses by *Gp*SPRY-414-2. Given the role of CLASP in the cytoskeleton, we investigated whether disruption of microtubules affected the ability of *Gp*SPRY-414-2 to suppress host defense responses. Application of the microtubule depolymerizing drug colchicine did not affect the cell death triggered by Gpa2/RBP-1 nor its suppression by the nematode effector. It is therefore possible that *Gp*SPRY-414-2 functions through a different pathway in regards to ETI suppression, independently from *St*CLASP. The results of colchicine treatment on ROS suppression assays are intriguing. Although the interaction between *Gp*SPRY-414-2 and *St*CLASP was not affected by the colchicine treatment, our data showed that after treatment with colchicine, no significant difference in ROS production was seen in leaves expressing *Gp*SPRY-414-2 compared to leaves expressing eGFP but in both cases ROS production was reduced compared to the production in *N. benthamiana* leaves expressing eGFP without colchicine treatment. In addition, *Gp*SPRY-414-2 alone significantly suppressed flg22-induced ROS production in absence of colchicine. The possibility remains that the nematode effector alters the function of CLASP and has an effect on the dynamics of the microtubule network which may be important for ROS signaling (Livanos et al., 2014). When adding colchicine to leaves expressing the effector, the microtubule network was already disturbed, so the effect of colchicine cannot be additive. Therefore, this could explain why no significant difference was observed. *Gp*SPRY-414-2 may therefore suppress flg22-induced ROS production by manipulating the microtubule network through CLASP. Consistent with a role for microtubules in ROS signaling, *map65* mutants with disturbed microtubule bundles have been shown to produce less ROS in response to flg22 and therefore to be more susceptible to *Pseudomonas syringae* (Guo et al., 2016).

We demonstrated that the *G. pallida* SPRYSEC effector directly interacts with *St*CLASP in yeast. Further evidence of the interaction of the two proteins *in planta* came from the BiFC assay that indicated the presence and co-localization of both the effector and its target in close vicinity. Moreover, we observed *in planta* that the potato target *St*CLASP was stabilized in the presence of *Gp*SPRY-414-2 and, conversely, that the effector seems to dimerize and maybe form oligomers in large protein complexes, which are promoted in the presence of *St*CLASP. The SPRY protein RanBPM (Ran-Binding Protein in the Microtubule-organizing center) also stabilizes its p73 target, by lowering E3-ligase-mediated ubiquitination and degradation (Kramer et al., 2005).

In *Gp*SPRY-414-2, the SPRY domain is the only protein feature identified and the main component of the effector, spanning about two thirds of the protein. SPRY domain-containing proteins are wide-spread among eukaryotes, including animals, plants, and fungi (Rhodes et al., 2005). These proteins are very diverse with overall no obvious relationship in terms of functions. However, the structure of the SPRY domain is highly conserved and it seems to provide an extremely versatile scaffold that mediates intermolecular protein-protein interactions. For example, the SPRY domain of RanBPM provides a platform for the interaction of several signaling components, besides binding to RAN in immune cells and in neurons (Murrin and Talbot, 2007). Similarly, the B30.2 domain, which consists of the combination of an N-terminal PRY and C-terminal SPRY sub-domains, is involved in heterotypic protein-protein interactions (Perfetto et al., 2013). For example, the SPRY domain of Pyrin (also known as TRIM20) binds to several inflammasome components thereby modulating their activity in human cells (Papin et al., 2007). In nematode SPRYSEC effector proteins, most amino acid sites under diversifying selection are located in loops at the surface of the protein, which constitute the hypervariable regions in the SPRY domains otherwise interspersed with conserved blocks of sequences (Rehman et al., 2009; Diaz-Granados et al., 2016). It was thus suggested that this hypervariable surface mediates the interaction between the SPRYSEC effectors and their host targets.

There is also mounting evidence of SPRY-containing proteins dimerizing and forming oligomers upon ligand binding. Several examples come from proteins that contain a B30.2 domain and that belong to the tripartite motif (TRIM) family. For instance, the cytosolic TRIM5α provides protection for mammalian cells against retroviral infection by intercepting the virus capsid cores. The TRIM5α SPRY domain directly binds the capsid but with low affinity. The system requires the dimerization of TRIM5α and the assembly of the dimers into a TRIM lattice in order to tailor the SPRY domain positioning and promote avid binding of the viral capsid (Roganowicz et al., 2017). The crystal structure of the TRIM20 coiled-coil/B30.2 fragment also showed clear formation of dimers, with the coiled-coil motifs serving as scaffold for the presentation of the globular B30.2 domains (Weinert et al., 2015). Similarly, *Gp*SPRY-414-2 may interact with *St*CLASP but needs to dimerize to properly bind its target and promote its stability. This may create a microtubule-anchored environment to further regulate protein shuttling between different cellular compartments.

### The multifaceted role of microtubules in plant–pathogen interactions

Microtubules are one of the three types of cytoskeleton elements in cells along with actin filaments and intermediate filaments. Microtubules are composed of alpha and beta tubulin dimers and play fundamental roles in a range of biological processes such as mitosis, cell migration, maintenance of cell shape, movement of cellular structures (Galjart, 2005; Akhmanova and Steinmetz, 2008), and more recently a role in plant defense has emerged (Guo et al., 2016; Quentin et al., 2016). Plant microtubules go through a range of reorganizations when plants are exposed to pathogens. As reviewed by Hardham (2013), pathogenic bacteria, fungi and oomycetes can induce a range of alterations in microtubule arrays and dynamics; viruses take advantages of microtubules to facilitate their movement and transmission; cyst and root-knot nematodes manipulate microtubules as part of the process of enhancing mitosis and partial cytokinesis during the development of their feeding sites. The microtubule is a polar tube with a slow-growing minus end and a fast-growing plus end and this leads to its most prevalent behavior called dynamic instability, a process where it grows and shrinks at a rapid but constant rate through polymerization and depolymerization of tubulins (Howard and Hyman, 2003; Horio and Murata, 2014). A functional microtubule network is essential for plant parasitic nematodes to establish in their host and hence *M. incognita* development was nearly completely inhibited in pea roots that were treated with colchicine early after infection (Wiggers et al., 2002). Regulation of the dynamic behavior of microtubules requires the cooperation of microtubule-associated proteins. For instance the MAP65/Ase1/PRC1 protein family is important for crosslinking anti-parallel microtubule bundles (Gaillard et al., 2008; Tulin et al., 2012). Consistent with the important role of this process in nematode feeding site development, the Arabidopsis *map65* mutant is less susceptible to *M. incognita* (Caillaud et al., 2008). *Map65* mutants are also less susceptible to infection with the oomycete *Hyaloperonospora arabidopsidis* and the ascomycete *Erysiphe cruciferarum* (Quentin et al., 2016). This lower susceptibility is correlated with constitutive activation of genes involved in salicylic acid biosynthesis, signaling, and defense.

CLASPs are conserved in animals, yeast, fungi and plants (Gardiner, 2013) and are important in maintaining the stability of microtubules (Mimori-Kiyosue et al., 2005). In Arabidopsis, the *clasp-1* mutant was reported to have reduced cell expansion, decreased microtubule polymerization and increased sensitivity to the microtubule-depolymerizing agent oryzalin (Ambrose et al., 2007). In addition, the *clasp-1* mutant displayed a range of auxin-related defects such as abundant lateral roots, reduced apical dominance as well as a reduction in root apical meristem size (Ambrose et al., 2007; Kirik et al., 2007). It was recently reported that CLASP promotes endocytic recycling of the auxin transporter PIN2 and restricts its degradation via direct interaction with SNX1, which is involved in maintaining PIN levels (Ambrose et al., 2013). Interestingly, it seems that plant parasitic nematodes have evolved to manipulate polarity shifts of PIN proteins to facilitate their establishment in the host plant. An enhanced auxin response was seen at the infection sites of both cyst and root-knot nematodes while auxin signaling mutants were shown to have significantly lower nematode infection (Goverse et al., 2000; Grunewald et al., 2009). It is therefore possible that the aberrant microtubules or auxin distribution in *clasp-1* mutants could influence nematode infection. Unfortunately, infection experiments on the Arabidospsis *clasp-1* mutant using the beet cyst nematode *Heterodera schachtii* were inconclusive (data not shown). Altogether, these findings, in conjunction with those described here, support a multifaceted role for the microtubules in the plant response to infection by nematodes and other pathogens.

## Sequences submitted to GenBank:

- *St*CLASP-Y2H = MH003297
- *Gp*SPRY-414-2 = MH003298
- *Gp*SPRY-24D4 = MH003299
- *Gp*SPRY-12N3 = MH003300
- *Gp*SPRY-17I9-1 = MH003301

## Author contributions

YM, SM, JJ, and GG conceived and designed the research that was coordinated by SM. Experiments were done by YM, SM, and KW with support from AH, LB, AD-G, and AG. Data were analyzed by YM, SM, JJ, and GG who also wrote the manuscript. All authors read and approved the final manuscript.

### Conflict of interest statement

The authors declare that the research was conducted in the absence of any commercial or financial relationships that could be construed as a potential conflict of interest.
