# Supplementary material for: The Globodera pallida SPRYSEC Effector GpSPRY-414-2 That Suppresses Plant Defenses Targets a Regulatory Component of the Dynamic Microtubule Network
Source: Front Plant Sci. 2018 Jul 12;9:1019. doi: 10.3389/fpls.2018.01019 (PMC6052128; doi:10.3389/fpls.2018.01019)
Supplement: Supplementary file 1 [file Table_1.pdf]

Primer name	Sequence 5’->3’	Primer use & Clone identity
SPRY24D4-F	ACCATGAATGAACAAAATGCATATGGTTTC	Cloning *GpSPRY-24D4*[1] Sequence related to GPLIN_001465500
SPRY24D4-R	TCAAATGCCATCGGCAAAGTT	
SPRY24D4-R3	AATGCCATCGGCAAAGTT	
SPRYGpE414-F	ACCATGTGGCCGCCAAAAACG	Cloning *GpSPRY-414-2* Sequence related to GPLIN_000195600
SPRYGpE414-R	TCATTTTTCAGTTTCTAAATTCCATTTG	
SPRYGpE414-R3	TTTTTCAGTTTCTAAATTCCATTTG	
GpE414F	GCTGTCTTCGCTGTTCAGTC	dsRNA synthesis *GpSPRY-414-2*
GpE414T7R	GTAATACGACTCACTATAGGGTTGCCGACACCATACCGT	
GpE414R	TTGCCGACACCATACCGT	
GpE414T7F	GTAATACGACTCACTATAGGGGCTGTCTTCGCTGTTCAGTC	
GpE414testF2	GGATGCGGCGTGGATTTAG	Semi-qRT-PCR *GpSPRY-414-2*
GpE414testR2	GGAAGTCCGCTCCAAAGTTC	
GpEF1α-F	AACATCTCTGTGAAGGACATTCG	Semi-qRT-PCR *GpEF1α*
GpEF1α-R	TCTCCTTAAGTTCGGCGAATTTGC	
GFPF	GCTGGAGTACAACTACAACT	dsRNA synthesis GFP control[2]
GFPT7R	GTAATACGACTCACTATAGGGGGCAGATTGCGTGGACAGGT	
GFPR	GGCAGATTGCGTGGACAGGT	
GFPT7F	GTAATACGACTCACTATAGGGGCTGGAGTACAACTACAACT	
G1-5-Cloning-For	ATGGAGGAGGCACTGGAAT	Cloning *St*CLASP Sequence related to scaffold PGSC0003DMB000000115
G1-5-Cloning-Rev	CTAACTGCGGTTAGCATCTATGG	
G1-5-Cloning-noStp-Rev	ACTGCGGTTAGCATCTATGG	
G1-5-PCR-F	GCTATCTACATTCTTACCTGCC	Testing potato cDNA for the presence of *St*CLASP
G1-5-PCR-R[3]	AACTTCTCATAAACAAACCTACAA	
G1-5-F1[4]	AAAGCCTGCTCAAAGGTCTG	Sequencing G1-5 and *StCLASP* constructs
G1-5-F2	GGGCCTAGAGGTTTTCCAGA	
G1-5-F3	CCCCTCGTATAGAAGTGGATTT	
G1-5-F2240	TGAACCAAGCATTCCTCAGA	
G1-5-F2477	AGATGCCATGGAGGATTCAG	
G1-5-880F	AGCCCAAAAATCCCTTAG	
G1-5-1600R	GCATCTCCTACACAACACTTT	
G1-5-1500F	CAAGATGCTGTGAGTATGC	
G1-5-2840R	ATGGGGCAAAATCCTCTC	

Primer name	Sequence 5’->3’	Primer use & Clone identity
M13-F(-20)	GTAAAACGACGGCCAG	Sequencing primers located on the cloning vectors
M13-R(-24)	AGGAAACAGCTATGACCATG	
pDEST32-BaitBD-F	AACCGAAGTGCGCCAAGTGTCTG	
pDEST22-PreyAD-F	TATAACGCGTTTGGAATCACT	
pDEST-R	AGCCGACAACCTTGATTGGAGAC	
p35S-FOR	AAGGAAGTTCATTTCATTTGGAGAGGA	
t35S-REV	CAACACATGAGCGAAACCCTATAAGAA	
pCL112-NYFP-F	CAACTACAACAGCCACAACG	
pCL113-CYFP-F	CCGACAACCACTACCTGAG	
C-mRFP-FOR2	CCTACAAGACCGACATCAAG	
N-mRFP-REV	TTCAAGTAGTCGGGGATGT	
Cterm-GFP-FOR	ACAACCACTACCTGAGCAC	
Nterm-GFP-REV	CGGACACGCTGAACTTG	

Supplementary Table 1: Sequences of primers used for cloning without signal peptide the effector genes *GpSPRY-414-2* and *GpSPRY-24D4*, as well as the *StCLASP*, and for sequencing all constructs. Artificial sequences added for cloning purposes are underlined when relevant (sequence leader and artificial stop codon sequences, as well as T7 promoter for double-stranded RNA synthesis used in nematode silencing).

(1) Primer from reference Mei *et al.* (2015). Only a small subset of the SPRY domain gene family in *Globodera pallida* is likely to encode effectors, two of which suppress host defences induced by the potato resistance gene *Gpa2*. *Nematology* 17, 409-424. [doi: 10.1163/15685411-00002875].

(2) Primer from reference Whisson *et al.* (2005). A method for double-stranded RNA-mediated transient gene silencing in *Phytophthora infestans*. *Molecular Plant Pathology* 6, 153-163. [doi: 10.1111/j.1364-3703.2005.00272.x].

(3) Primer located in the 3’UTR region of the gene based on the potato yeast two-hybrid G1-5 prey clone sequence.

(4) Primer originally designed based on the yeast two-hybrid prey clone G1-5 but that imperfectly matches the sequence present in the full-length *StCLASP* clone.
