# Supplementary material for: The Globodera pallida SPRYSEC Effector GpSPRY-414-2 That Suppresses Plant Defenses Targets a Regulatory Component of the Dynamic Microtubule Network
Source: Front Plant Sci. 2018 Jul 12;9:1019. doi: 10.3389/fpls.2018.01019 (PMC6052128; doi:10.3389/fpls.2018.01019)
Supplement: Supplementary file 3 [file Presentation_1.pdf]

Supplementary Material

The *Globodera pallida* SPRYSEC Effector *Gp*SPRY-414-2 that Suppresses Plant Defenses Targets a Regulatory Component of the Dynamic Microtubule Network

Yuanyuan Mei, Kathryn Mary Wright, Annelies Haegeman, Lander Bauters, Amalia Diaz-Granados, Aska Goverse, Godelieve Gheysen, John Trefor Jones and Sophie Mantelin*

*** Correspondence:** sophie.mantelin@hutton.ac.uk

Supplementary Figure S1: SPRYSEC candidate effectors and related protein sequences. Amino acid sequence alignments of the effector proteins without signal peptide. **(A)** *Gp*SPRY-414-2 clone with *Globodera pallida* related best BLAST hit sequence GPLIN_000195600. **(B)** *Gp*SPRY-24D4 with *G. pallida* related best BLAST hit sequence GPLIN_001465500 and the expressed sequence tag (EST GenBank accession FJ810124) originally used as template to design the cloning primers. The blue letters indicate amino acids conserved for all sequences in each alignment and red letters indicate amino acids at position under diversification. The SPRY domains predicted by SMART analysis are underlined under the consensus sequences. BLAST, Basic Local Alignment Search Tool; EST, Expressed Sequence Tag; SMART, Simple Modular Architecture Research Tool; SPRY, domain found in SP1a and Ryanodine receptors; SPRYSEC, SPRY domain-containing protein predicted to be secreted.

EST (FJ810124) reference: Jones et al. (2009). Identification and functional characterization of effectors in expressed sequence tags from various life cycle stages of the potato cyst nematode *Globodera pallida*. *Mol. Plant Pathol.* 10, 815-828. doi: 10.1111/j.1364-3703.2009.00585.x

Supplementary Figure S2: *St*CLASP clone and related protein sequences. Amino acid sequence alignment of full-length and yeast two-hybrid-related potato CLASP clones (*St*CLASP-FL and *St*CLASP-Y2H, respectively) with both homologous CLASP sequences found in the genome of potato (XP_006350293.1 and XP_015159473.1) and tomato (Solyc09g063030 and Solyc06g008040). The 4 TOG domains (black) and the 2 CLASP-N regions (green) predicted with confidence by SMART analysis are underlined under the consensus sequence. The blue letters indicate amino acids conserved in more than 80% of the aligned sequences, red letters indicate the most prevalent amino acids present at positions under diversification and orange letters indicate amino acids otherwise present at these variable locations. CLASP, Cytoplasmic Linker Protein

(CLIP)-Associated Protein; SMART, Simple Modular Architecture Research Tool; TOG, Tubulin-binding Tumor Overexpressed Gene.

Supplementary Figure S3: Both *Globodera pallida* effectors *Gp*SPRY-414-2 and *Gp*SPRY-24D4 as well as the full-length *St*CLASP protein show a conserved localization pattern, irrespective of the tag position in the fusion proteins. Proteins tagged at the C-terminus with mRFP were transiently expressed in *Nicotiana benthamiana* leaves in combination with N-terminus GFP-tagged proteins. **(A-D)** N- and C-terminally tagged effector constructs co-expressed: maximum intensity projection (A) and single section through the nucleus (B) for *Gp*SPRY-414-2; similarly, maximum intensity projection (C) and single section through the nucleus (D) for *Gp*SPRY-24D4. Series presented with GFP in sub-panel 1, RFP in sub-panel 2 and an overlaid image in sub-panel 3. **(E)** Maximum intensity projection for the *St*CLASP co-expressed in the transgenic *N. benthamiana* line expressing a GFP-tagged tubulin marker (TUA6). Series presented with GFP in sub-panel 1, RFP in sub-panel 2 and an overlaid image in sub-panel 3. **(F-H)** Free eGFP expressed on its own as control is a small cytoplasmic protein (without nuclear localization signal) that can passively diffuse into the nucleus. Maximum intensity projection at the cellular level (F) or focused on the cytoplasm (G), and single section through the nucleus (H). The pictures were all taken 2 days post infiltration by confocal microscopy. The GFP and RFP signals are displayed in green and magenta, respectively. Autofluorescence from chloroplasts is displayed in blue. Scale bars in A, C and F represent 50µm and in B, D, E, G and H represent 10µm. Each localization experiment was replicated at least twice.

Supplementary Figure S4: Split-YFP proteins detection by Western blot. Immuno-detection after SDS-PAGE and transfer of the split-YFP-tagged proteins used in bimolecular fluorescence complementation (BiFC) assay. *Globodera pallida* candidate effector genes *GpSPRY-414-2* and *GpSPRY-24D4* were transiently expressed in *Nicotiana benthamiana* leaves either alone or in combination with the *StCLASP* protein constructs (full length *StCLASP-FL* or truncated *StCLASP-Y2H*). The YFPn::*St*CLASP and YFPc-effector fusion proteins were detected using the polyclonal antibody anti-GFP (α-GFP sc8334). This is the full blot presented in Figure 6 after long exposure. The total amount of protein loaded per sample is represented in the Ponceau red panels for a section of the blot. Expected protein sizes for each fusion are indicated on the right hand side of the Western blot.

Supplementary Figure S5: Variation of flg22-induced ROS production in *N. benthamiana* in presence of control effector constructs. Reactive oxygen species (ROS) production induced by flg22 (100nM) in *Nicotiana benthamiana* leaves expressing eGFP, the nematode effector construct eGFP::*Gp*SPRY-24D4 or the aphid effector MP10 (Bos et al. 2010). ROS production is shown as total relative light units (RLU) over 60 minutes following elicitation. Values indicated are average RLU ± SE of 16 leaf disc samples with significant difference of means (Student's *t*-test at $P < 0.05$) indicated by an asterisk for the comparison between the eGFP and the effector samples (MP10 being

a suppressor of the flg22-mediated ROS production response while the nematode effector construct eGFP::*Gp*SPRY-414-2 has no effect).

Reference Bos et al. (2010). A functional genomics approach identifies candidate effectors from the aphid species *Myzus persicae* (green peach aphid). PLoS Genetics 6, e1001216. doi: 10.1371/journal.pgen.1001216.

Supplementary Table 1: Primers used for cloning without signal peptide the effector genes *GpSPRY-414-2* and *GpSPRY-24D4*, as well as the full-length *StCLASP*, and for sequencing all constructs. Artificial sequences added for cloning purposes are underlined when relevant (sequence leader and artificial stop codon sequences, as well as T7 promoter for double-stranded RNA synthesis used in nematode silencing).

(1) Primer from reference Mei et al. (2015). Only a small subset of the SPRY domain gene family in *Globodera pallida* is likely to encode effectors, two of which suppress host defenses induced by the potato resistance gene *Gpa2*. *Nematology* 17, 409-424. doi: 10.1163/15685411-00002875.

(2) Primer from reference Whisson et al. (2005). A method for double-stranded RNA-mediated transient gene silencing in *Phytophthora infestans*. *Mol. Plant Pathol.* 6, 153-163. doi: 10.1111/j.1364-3703.2005.00272.x.

(3) Primer located in the 3’UTR region of the gene based on the potato yeast two-hybrid G1-5 prey clone sequence.

(4) Primer originally designed based on the yeast two-hybrid prey clone G1-5 but that imperfectly matches the sequence present in the full-length *StCLASP* clone.

Supplementary Table 2: Prey clones recovered from the original yeast two-hybrid screen using *Gp*SPRY-414-2 as bait.

(1) Prey clone sequences correspond to partial sequencing of the insert in pDEST22 from the Gal4 fusion (vector and prey library sub-cloning sequences trimmed off).

(2) DNA hits in Potato genomes are based on the SOL source CDS PGSC DM v3.4 CDS except for the CLASP-related clones for which BLAST results are based on the SOL source CDS PGSC DM v3 scaffolds.
