# Supplementary figures and images for: The Globodera pallida SPRYSEC Effector GpSPRY-414-2 That Suppresses Plant Defenses Targets a Regulatory Component of the Dynamic Microtubule Network
Source: Front Plant Sci. 2018 Jul 12;9:1019. doi: 10.3389/fpls.2018.01019 (PMC6052128; doi:10.3389/fpls.2018.01019)

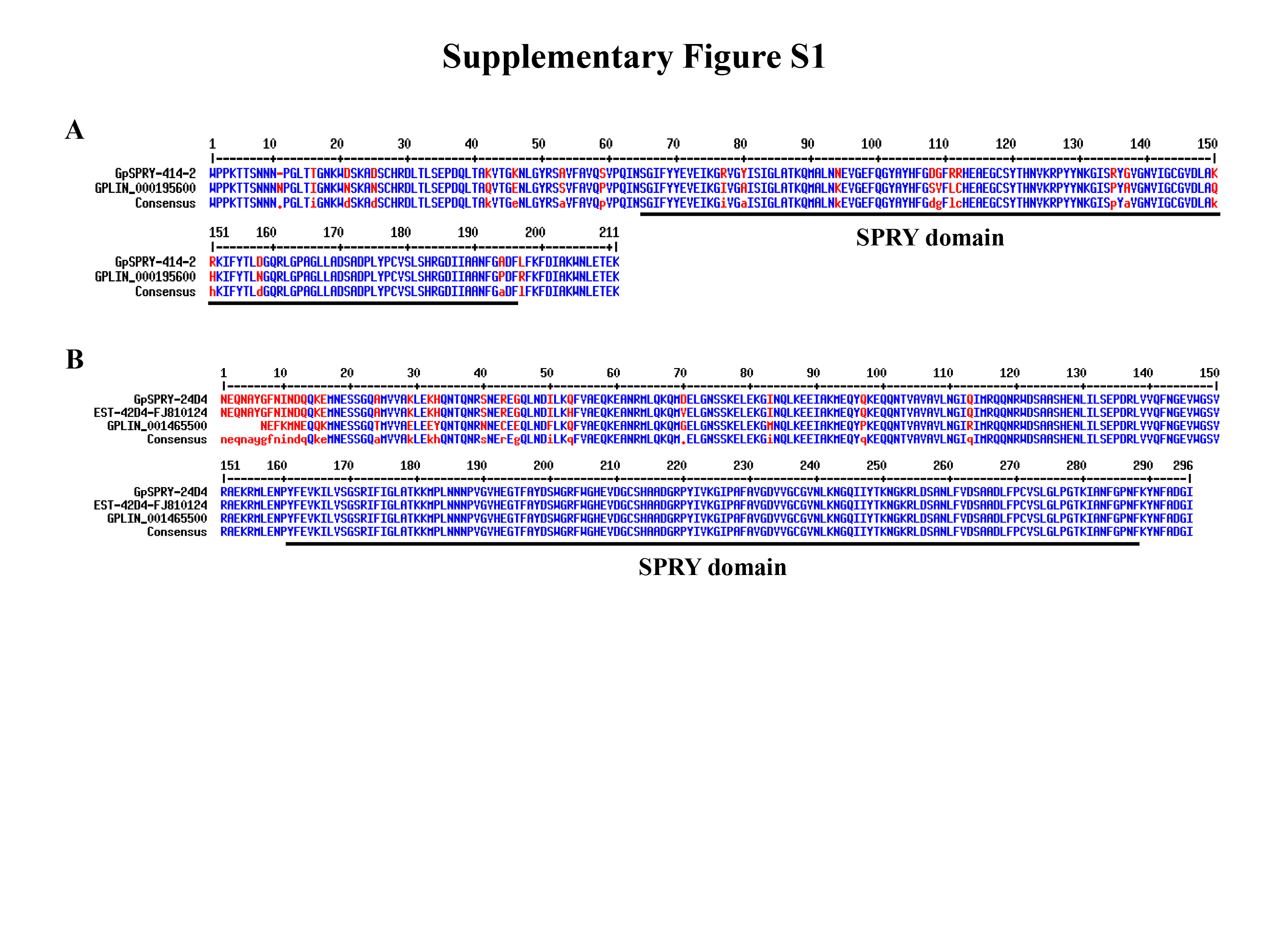

Supplement: Supplementary file 4 [file Image_1.JPEG]

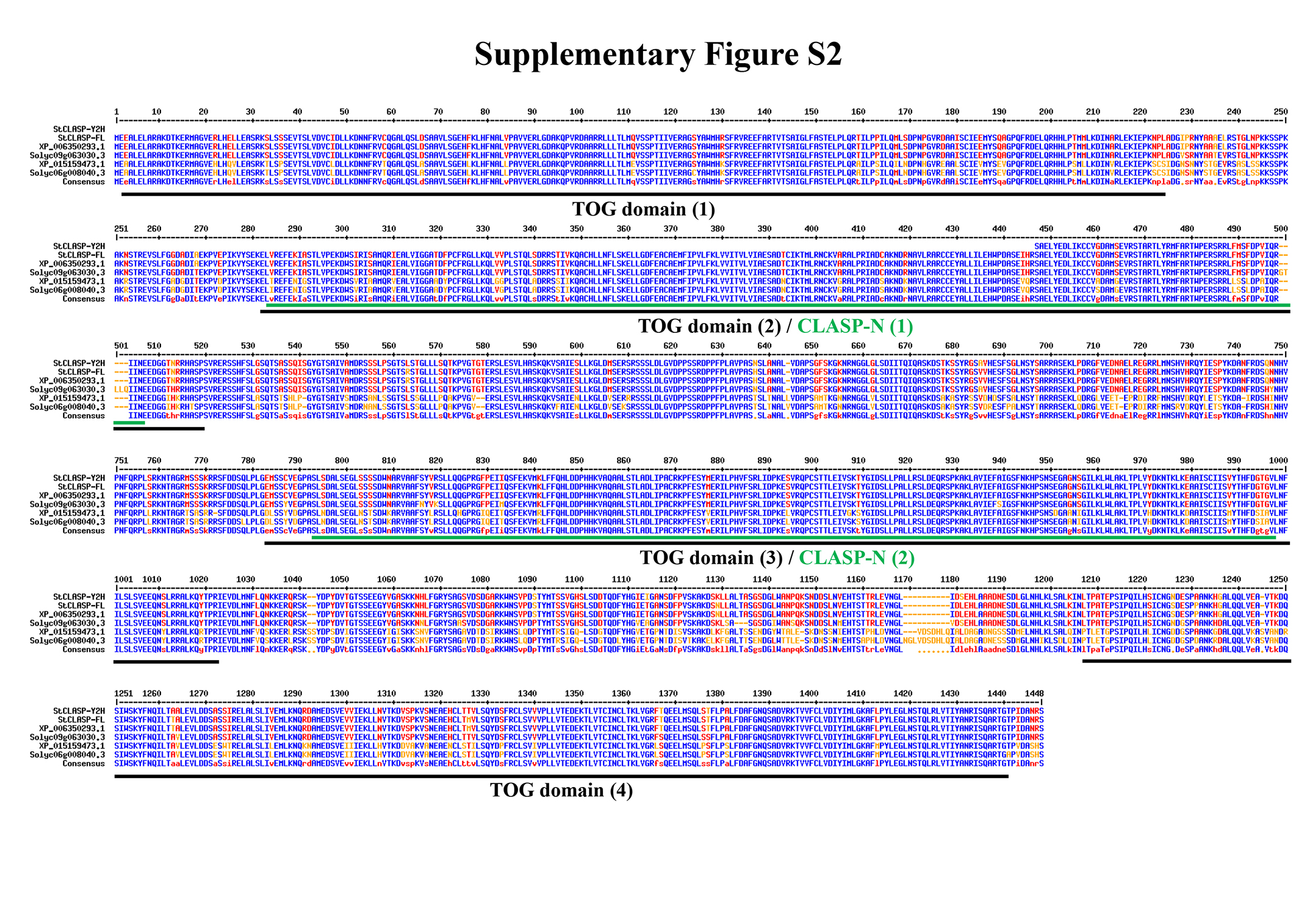

Supplement: Supplementary file 5 [file Image_2.JPEG]

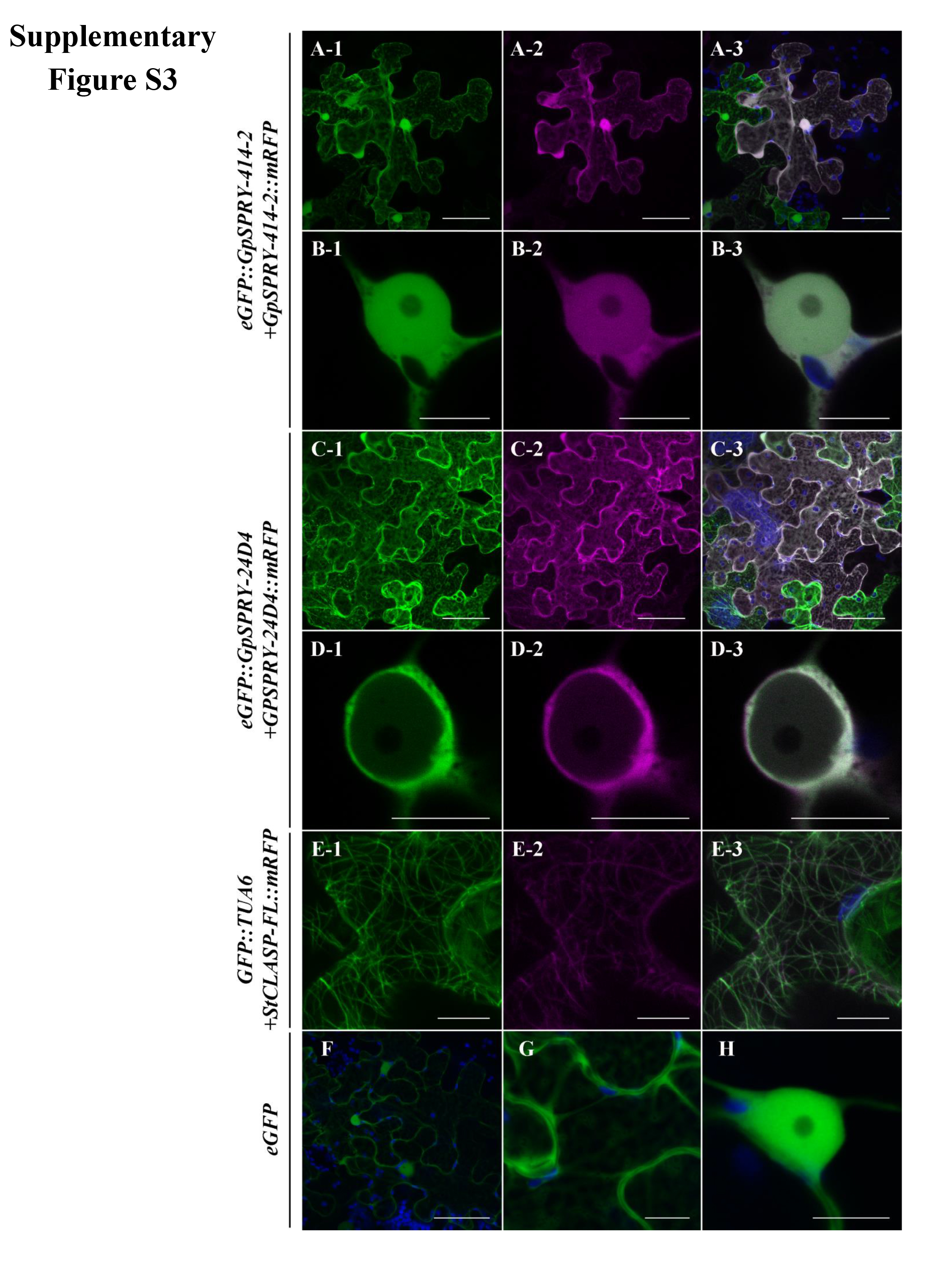

Supplement: Supplementary file 6 [file Image_3.JPEG]

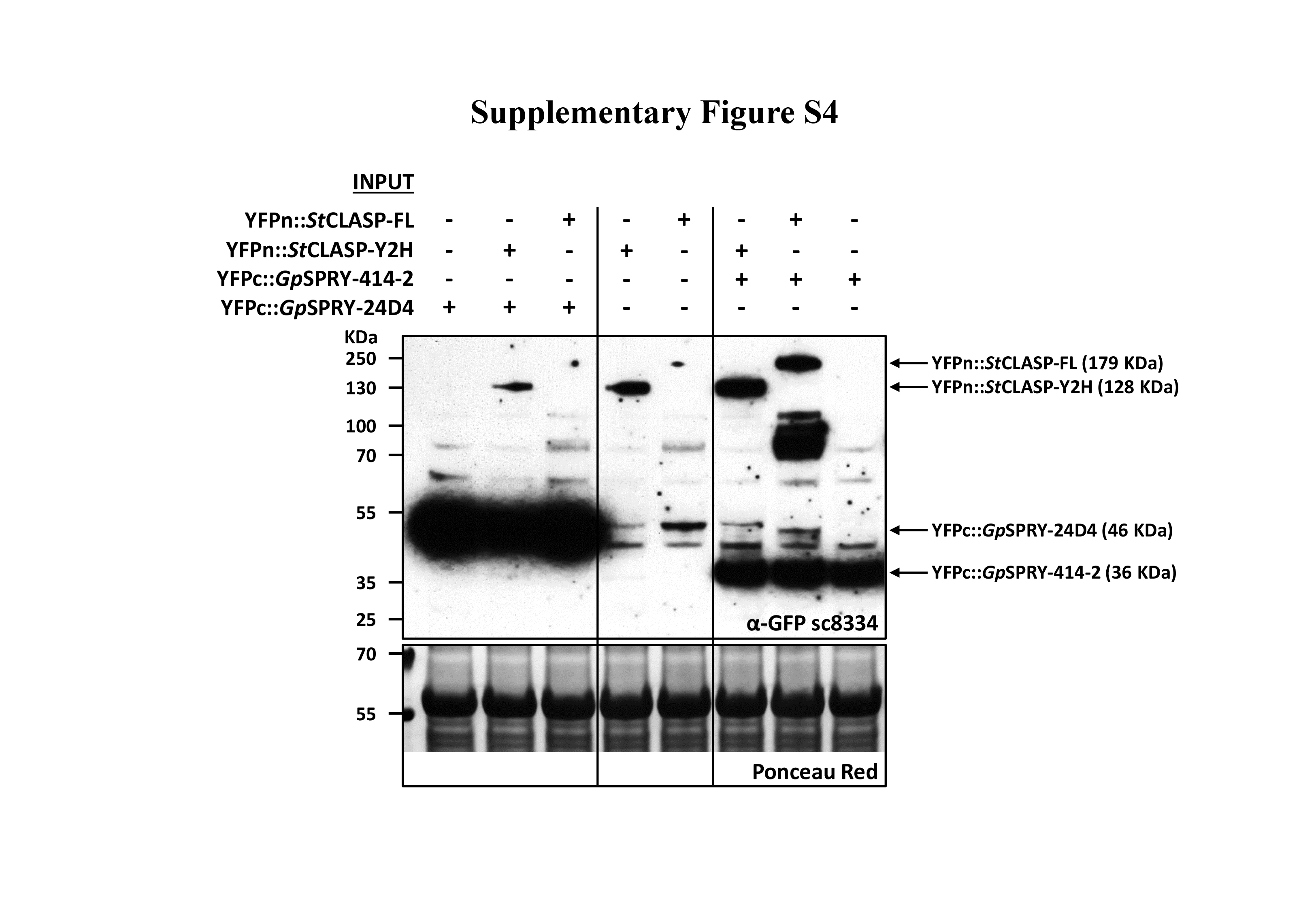

Supplement: Supplementary file 7 [file Image_4.JPEG]

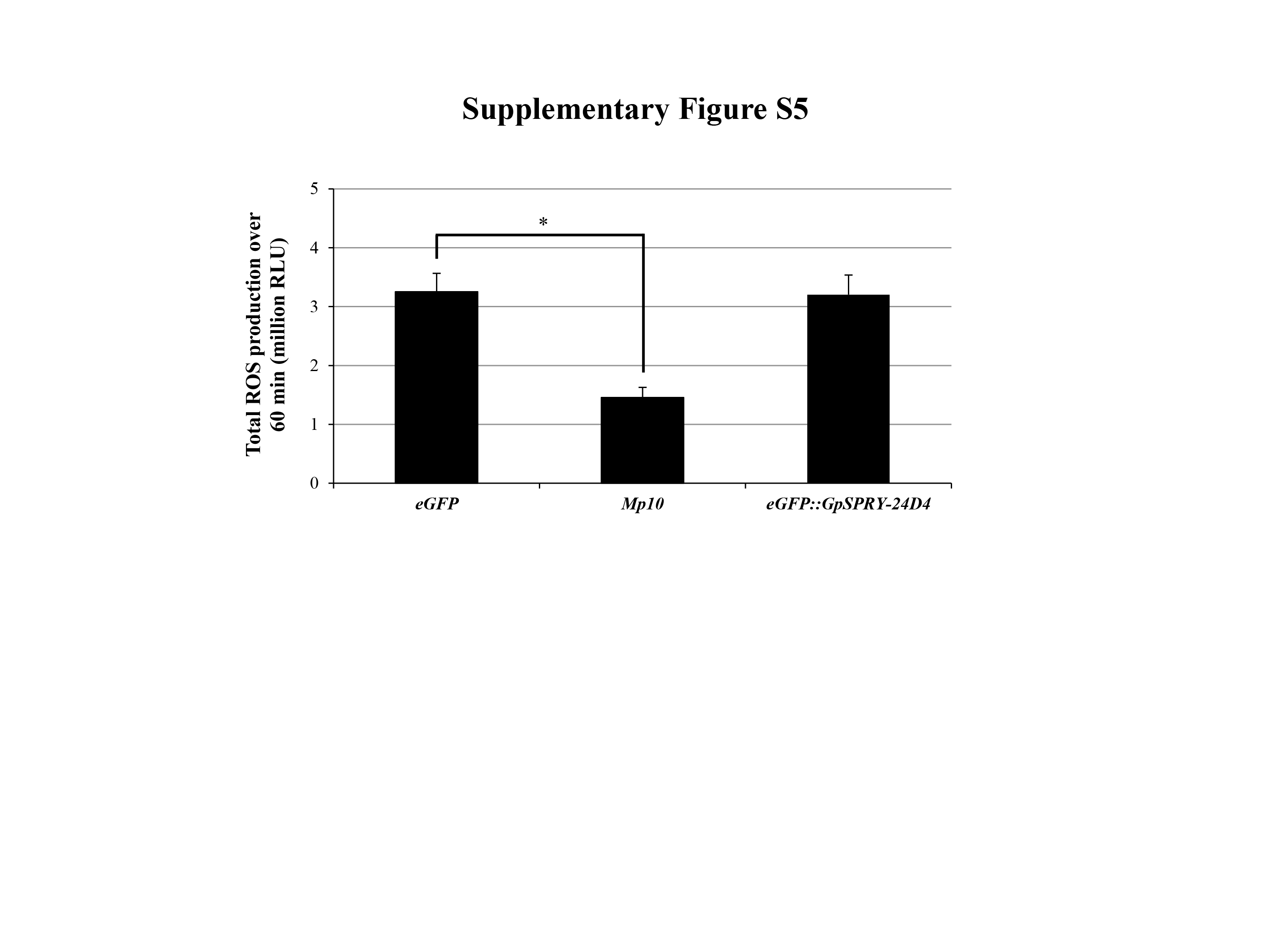

Supplement: Supplementary file 8 [file Image_5.JPEG]
